# Low Expression of miR-20a-5p Predicts Benefit to Bevacizumab in Metastatic Breast Cancer Patients Treated within the TANIA Phase III Trial

**DOI:** 10.3390/jcm9061663

**Published:** 2020-06-01

**Authors:** Gabriel Rinnerthaler, Simon Peter Gampenrieder, Hubert Hackl, Markus Steiner, Claudia Monzo-Fuentes, Thomas Melchardt, Teresa Magnes, Florian Huemer, Theresa Westphal, Clemens Hufnagl, Cornelia Hauser-Kronberger, Alexander Egle, Richard Greil

**Affiliations:** 1 Laboratory for Immunological and Molecular Cancer Research (SCRI-LIMCR), Medical Oncology, Haemostaseology, Infectiology and Rheumatology, Department of Internal Medicine III with Haematology, Salzburg Cancer Research Institute, Oncologic Center Salzburg, Paracelsus Medical University Salzburg, 5020 Salzburg, Austria; g.rinnerthaler@salk.at (G.R.); s.gampenrieder@salk.at (S.P.G.); mark.steiner@salk.at (M.S.); c.monzo-fuentes@salk.at (C.M.-F.); t.melchardt@salk.at (T.M.); t.magnes@salk.at (T.M.); f.huemer@salk.at (F.H.); theresa.westphal@googlemail.com (T.W.); cl.hufnagl@crcs.at (C.H.); a.egle@salk.at (A.E.); 2 Cancer Cluster Salzburg, 5020 Salzburg, Austria; 3 Institute of Bioinformatics, Biocenter, Medical University of Innsbruck, 6020 Innsbruck, Austria; hubert.hackl@i-med.ac.at; 4 Department of Pathology, Paracelsus Medical University Salzburg, 5020 Salzburg, Austria; c.kronberger@salk.at

**Keywords:** breast cancer, microRNA, miR-20a-5p, bevacizumab, predictive biomarker

## Abstract

Background: In metastatic breast cancer (MBC) patients, no biomarker predicting benefit to a bevacizumab-containing therapy has been established yet. MicroRNAs (miRNAs) are involved in angiogenesis and treatment resistance and therefore could be of predictive value. Methods: Profiling of 754 miRNAs was performed in tumor samples of 58 MBC patients treated with a bevacizumab-containing first-line regimen (learning set). Based on progression-free survival (PFS), patients were divided into responders (R) and non-responders (NR). Differentially expressed miRNAs between R and NR were analyzed in a cohort of 57 patients treated with first-line chemotherapy without bevacizumab (control set), to exclude miRNAs providing prognostic information. MiRNA candidates significantly associated with PFS in multivariate analysis were further validated in tumor samples of 203 patients treated within the phase III trial TANIA randomizing between chemotherapy either alone or with bevacizumab after progression on first-line bevacizumab. Results: Low expression of miR-20a-5p (multivariate *p* = 0.035) and miR-21-5p (multivariate *p* = 0.004) were significantly associated with longer PFS in the learning set, but not in the control set. In samples from the TANIA trial, low expression of miR-20a-5p was also significantly associated with longer PFS (hazard ration (HR) 0.60; 95%-CI 0.37–0.89; *p* = 0.012) and longer overall survival (OS; HR 0.54; 95%-CI 0.32–0.83; *p* = 0.007) in the bevacizumab arm but not in the chemotherapy-only arm (PFS: HR 0.73, *p* = 0.119; OS: HR 1.01; *p* = 0.964). For miR-21-5p no significant association with PFS or OS in both treatment arms was observed. Conclusion: MiR-20a-5p expression in breast cancer tissue was predictive for a greater benefit from bevacizumab-containing therapy in two independent cohorts.

## 1. Introduction

Metastatic breast cancer (MBC) is defined as a stage of breast cancer with detectable macro metastasis at distant organ sites despite locoregional lymph nodes. MBC is still virtually an incurable disease and is worldwide the most common cause of cancer mortality in women [1,2]. Prognosis and treatment options clearly depend on the molecular subtype determined either by immunohistochemistry or molecular assays as well as on proliferation rate. By the introduction of novel endocrine agents, targeted agents and immunotherapy, a substantial progress has been made for both hormone-receptor-positive as well as human epidermal growth factor receptor 2 (HER2/neu) positive metastatic breast cancers [3]. Triple-negative breast cancer (TNBC), a heterogeneous breast cancer subgroup defined by the absence of estrogen receptor, progesterone receptor and HER2/neu expression, is associated with the worst prognosis.

Based on a strong rationale for anti vascular endothelial growth factor (VEGF) treatment in metastatic breast cancer (MBC) and promising preclinical data, great hopes have been placed on the anti-VEGF antibody bevacizumab. Clinical phase III trials, however, reported conflicting results. In HER2-negative MBC, the addition of bevacizumab to standard first-line chemotherapy consistently improved progression-free survival (PFS), however, the extent of PFS improvement varied between the studies and no improvement in overall survival was shown [4,5,6,7]. Unfortunately, biomarkers predicting benefit from a bevacizumab containing treatment are still lacking. Promising biomarkers like plasma levels of VEGF-A or vascular endothelial growth factor receptor 2 (VEGFR-2) [7,8], tissue markers like the VEGFR co-receptor neuropilin-1 (NRP-1) [9,10,11], single nucleotide polymorphisms (SNPs) in VEGF-A [12], deoxyribonucleic acid (DNA) methylation signatures [13] or clinical markers like treatment-induced hypertension [12,14,15] failed to demonstrate clinical utility or reproducibility.

MicroRNAs (miRNA) are small non-coding, single-stranded ribonucleic acid (RNAs) regulating gene expression at a posttranscriptional level. Besides various physiological functions, miRNAs are known to be involved in tumor evolution [16]. Differentially expression of numerous miRNAs contribute to specific processes associated with breast cancer invasiveness and stemness [17]. Furthermore, miRNAs contribute to the regulation of angiogenesis [18,19] and development of treatment resistance [20,21]. Recently it has been shown, that a miR-124 mimic treatment can reverse drug sensitivity in doxorubicin as well as in paclitaxel resistant MCF7 cells [22,23]. In colorectal cancer, where anti-VEGF treatments are an integral part of advanced disease treatment, the role of angioregulatory miRNAs has been investigated in a more detail, as recently reviewed by Soheilifar et al. [24]. Due to the numerous functions, miRNA expressions are also of prognostic importance. For example, miR-124 and miR-126 relative expressions have been shown to be significantly lower in breast cancer tissue compared to corresponding tumor adjacent normal tissue and lower expression levels further were associated with worse clinicopathological parameters [25].

In this retrospective analysis, we screened for miRNAs predicting a benefit from the addition of bevacizumab to first-line chemotherapy in patients with HER2-negative MBC treated at our institution. A control cohort of patients treated with chemotherapy alone was used to exclude miRNAs providing prognostic information only. Promising candidates were finally validated in tissue samples from patients treated within the multicenter, open-label, randomized phase III trial TANIA (NCT01250379) (Figure S1). In this trial the addition of bevacizumab to second- and third-line chemotherapy (CT) significantly improved second-line PFS (primary endpoint) in patients with bevacizumab-pretreated locally recurrent or MBC (Hazard Radio (HR) 0.75; 95% CI 0.61–0.93; *p* = 0.0068) [26]. No significant differences in third-line PFS and overall survival (OS), the secondary endpoints, were observed [27].

## 2. Experimental Section

### 2.1. Patients and Study Design

#### 2.1.1. Screening Cohort

Patients with MBC treated at our tertiary cancer center between 2006 and 2012 were screened using a comprehensive patient database and 115 patients treated with first-line chemotherapy with (learning set; *n* = 58) or without bevacizumab (control set; *n* = 57) were identified. Key inclusion criteria were histologically confirmed adenocarcinoma of the breast, locally advanced inoperable or metastatic tumor stage, Eastern Cooperative Oncology Group (ECOG) performance status 0–3, at least one chemotherapy line for advanced disease, sufficient medical records allowing calculation of PFS and OS and sufficient tumor material for RNA isolation (yielding at least 1 µg of total RNA). Based on the median overall PFS, patients were divided into a responder (R) and a non-responder group (NR). PFS was defined as time from treatment initiation until progression or death from any cause, whichever occurred first. Formalin-fixed paraffin-embedded (FFPE) tissue blocks containing samples from primary tumors (72%), or if available, from metastatic sites (28%), were selected by an experienced breast pathologist. All tissue samples were collected prior to the start of first-line chemotherapy for metastatic disease. Details on patient characteristics and tumor material of the screening cohort are provided in Table 1.

#### 2.1.2. Validation Cohort

The study design of the TANIA phase III trial is summarized in Figure S1. RNA samples from 203 patients consenting to optional translational research were retrospectively analyzed. A total of 98 patients were treated with chemotherapy plus bevacizumab and 105 patients with chemotherapy alone. RNA was isolated from archival primary or metastatic FFPE tumor samples collected before study entry. The majority of samples (89.4%) were obtained from the primary tumor.

### 2.2. MiRNA Expression Analysis

#### 2.2.1. Screening Cohort-Learning Set

Total RNA was purified from FFPE-tissue using the *mir*Vana^TM^ miRNA Isolation Kit from Ambion^®^ (Austin, TX, United States) and 1 µg was reversely transcribed to complementary DNA (cDNA) using the TaqMan^®®^ Reverse Transcriptase Kit (Applied Biosystems^®^, Waltham, Massachusetts, USA) according to the manufacturer’s instructions. TaqMan Human MicroRNA array A and B Cards Set v3.0 (Applied Biosystems^®^, Waltham, Massachusetts, USA) was used to quantify the expression of 754 human miRNAs in the bevacizumab cohort. The experimenter was blinded regarding PFS. Expression levels (cycle threshold [C_T_]-values) were averaged over two replicates and normalized to miR-16-5p as endogenous control (ΔC_T_), which was identified as the most stable-expressed housekeeping tissue miRNA [28]. Differential expression between groups was based on the ΔΔC_T_-method. Only those miRNAs were considered with C_T_ < 40 in more than a quarter of patients (≥15 patients) and showing an interquartile range for ΔC_T_ > 0.5 over all patients.

#### 2.2.2. Screening Cohort-Control Set

Expression levels of potentially predictive microRNAs, selected in the bevacizumab-treated learning set, were analyzed by real-time quantitative polymerase chain reaction (qPCR) in the control set. Each miRNA was analyzed twice and identically processed as for screening.

#### 2.2.3. Validation Cohort

Isolated total RNA was provided by Roche^®®^. TaqMan Human MicroRNA array Custom Cards (Applied Biosystems^®^, Waltham, Massachusetts, USA) were used to quantify the expression of the selected miRNAs from screening. Again, expression levels were averaged over two replicates and normalized to miR-16-5p.

### 2.3. Statistical Analysis

#### 2.3.1. Screening Cohort

Differentially expressed miRNAs between R and NR were identified using moderated t-test. *p* values were adjusted for multiple testing based on the false discovery rate (FDR) according to the Benjamini-Hochberg method. In order to identify microRNAs which could contribute to the prediction of responders a regularized multivariate logistic regression classification was performed using Least Absolute Selection and Shrinkage Operator (LASSO) [29] including microRNAs with adjusted *p* < 0.2. To avoid over-fitting a 100 times five-fold cross validation procedure was performed based on the maximal area under curve (AUC). MicroRNAs with a more than two-fold change expression and adjusted *p* values < 0.1 and/or microRNAs which were included in the regression model more than five times out of the 100 iterations were selected for further analyses.

The association of miRNA expression, dichotomized based on median expression, with PFS or OS was analyzed for both the learning set and the control set using a log rank test. Survival curves were estimated by the Kaplan-Meier estimator. A multivariate analysis for each of the selected microRNAs was performed using Cox regression including widely accepted and documented clinical risk factors [30] disease-free interval (DFI ≤ 24 months vs. DFI > 24 months vs. de novo metastatic), adjuvant chemotherapy (yes vs. no), ECOG performance score (0–1 vs. ≥ 2), histologic subtype (ductal vs. lobular vs. others), tumor grade (1–2 vs. 3), receptor status (hormone receptor positive/HER2 negative vs. HER2 positive vs. triple-negative), and location of metastases (visceral vs. non-visceral) as categorical variables.

#### 2.3.2. Validation Cohort

In the TANIA samples the association of miRNA expression, based on median expression from the screening cohort, with PFS or OS was analyzed using a log rank test as well. Survival curves were estimated by the Kaplan-Meier estimator. Multivariate Cox regression analyses on PFS and OS included the trial stratification factors: first-line PFS (≥ 6 months vs. < 6 months), chemotherapy backbone (taxane vs. non-taxane vs. vinorelbine), actate dehydrogenase (LDH) level (> 1.5 vs. ≤ 1.5 the upper limit of normal), and hormone receptor status (triple negative vs. HR-positive) and including the following clinicopathological factors: DFI (≤ 24 months vs. > 24 months vs. de novo metastatic), histology (lobular + other vs. ductal), and location of metastases (visceral vs. non-visceral). The interaction term between dichotomized microRNA expression and treatment were tested in a Cox regression model. All statistical analyses were performed using the statistical software environment R version 3.5.2 (packages limma, glmnet, survival, ROCR).

### 2.4. Endpoints

Progression-free survival (PFS) was defined as time from treatment initiation until progression or death from any cause, whichever occurred first. Overall survival (OS) was defined as time from treatment initiation until death from any cause. Patients alive (for OS) and who had not experienced progression (for PFS) at the data cutoff date, were censored at the last follow-up date.

### 2.5. Ethics Approval and Consent to Participate

All patients included in the TANIA trial provided written informed consent including post hoc translational research. The protocol and all modifications were approved by independent ethics committees at all participating sites. The translational research study was approved by the ethics committee of the provincial government of Salzburg, Austria (IRB number: 415-E/2199/4-2017).

## 3. Results

### 3.1. Screening Cohort

The screening cohort consisted of 115 patients with MBC fulfilling the inclusion criteria, who received first-line chemotherapy with (learning set; *n* = 58) or without bevacizumab (control set; *n* = 57) at our institution between 2006 and 2012 (patient characteristics are outlined in Table 1). At a median follow-up of 27.3 months (range 1.5–89.0 months) in the learning set and 25.6 months (range 1.1–144.2 months) in the control set, median PFS was 10.9 and 11.8 months, respectively (HR 1.00; 95% CI 0.68–1.48; log-rank *p* = 0.995).

A total of eight miRNAs (miR-19b-3p, miR-21-5p, miR-9-5p, miR-590-5p, miR-106b-5p, miR-20a-5p, miR-19a-3p, and miR-27a-3p) were expressed at significantly different levels between responders (R) and non-responders (NR) (adjusted *p* < 0.1 in the learning set) (Table S1). All of them showed lower expression levels in R (Figures S2 and S3). An additional four miRNAs (miR-210-3p, miR-28-5p, miR-155-5p, and miR-224-5p) were selected based on a regularized logistic regression classification model, a method previously applied to develop prognostic and predictive microRNA signatures [31]. These additional selected microRNAs were not significantly differential expressed (0.2 > adjusted *p* > 0.1) but substantially contribute to the optimal separation between R and NR as they were repeatedly (> 5 times) included in the optimal regularized classification model from a 100 times five-fold cross validation procedure (Table 2).

**Figure 1:**
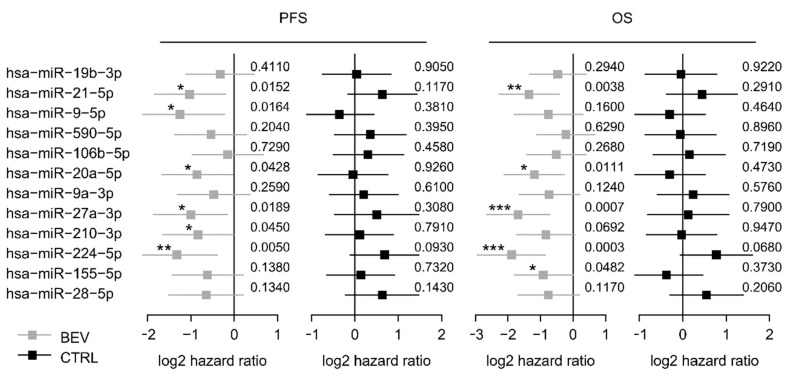
Forest plots for PFS and OS based on the median expression of the selected miRNAs in the screening cohort. Log 2-hazard ratio (HR) and 95%-CI (forest plots) for PFS and OS based on the median expression of the selected miRNAs in the bevacizumab treated group (learning set) and the median expression in the control group treated with chemotherapy alone. (*** *p* < 0.001, ** 0.001 ≤ *p* < 0.01, * 0.01 ≤ *p* < 0.05; exact *p* values on the right). BEV: bevacizumab, CTRL: control

These 12 selected miRNAs from the screening set were analyzed in the control set (Table S1). Among these, six miRNAs (miR-9-5p, miR-20a-5p, miR-21-5p, miR-27a-3p, miR-210-3p, and miR-224-5p) were significantly associated with PFS in the bevacizumab cohort but not, or conversely, in the control cohort, suggesting a predictive value for bevacizumab response (Figure 1). Kaplan-Meier curves of all 12 miRNAs are provided in the supplement (Figure S4). Furthermore, five miRNAs were associated with OS in the learning set but not in the control set including miR-20a-5p and miR-21-5p (Figure 1 and Figure S5). Each of the 12 miRNA was included in a multivariate analysis together with trial stratification and clinicopathological factors. MiR-20a-5p and miR-21-5p remained independent predictors for shorter PFS in this multivariate analysis (*p* = 0.004 and *p* = 0.035, respectively; Table S2) and were therefore selected for further validation.

### 3.2. Validation Cohort

The validation cohort consisted of 203 patients treated within the prospective phase III trial TANIA. Patient characteristics in the biomarker-population were broadly representative of those in the intent-to-treat (ITT) population of the TANIA trial (Table S3). The benefit from adding bevacizumab to standard chemotherapy seemed lower in the biomarker cohort compared to the ITT population (HR for PFS 0.86 vs. 0.75) and the median PFS values were slightly lower in both treatment arms (Table S4). There was no difference in OS in both cohorts between the two study arms (HR 0.95 and HR 0.96; Table S4).

Expression levels of miR-20a-5p and miR-21-5p in the TANIA trial were defined based on the median expression levels in the learning set. High expression of miR-20a-5p was defined as ΔC_T_ ≤ 3.775 and low expression as ΔC_T_ > 3.775. High expression of miR-21-5p was defined as ΔC_T_ ≤ 2.080 and low expression as ΔC_T_ > 2.080.

**Figure 2:**
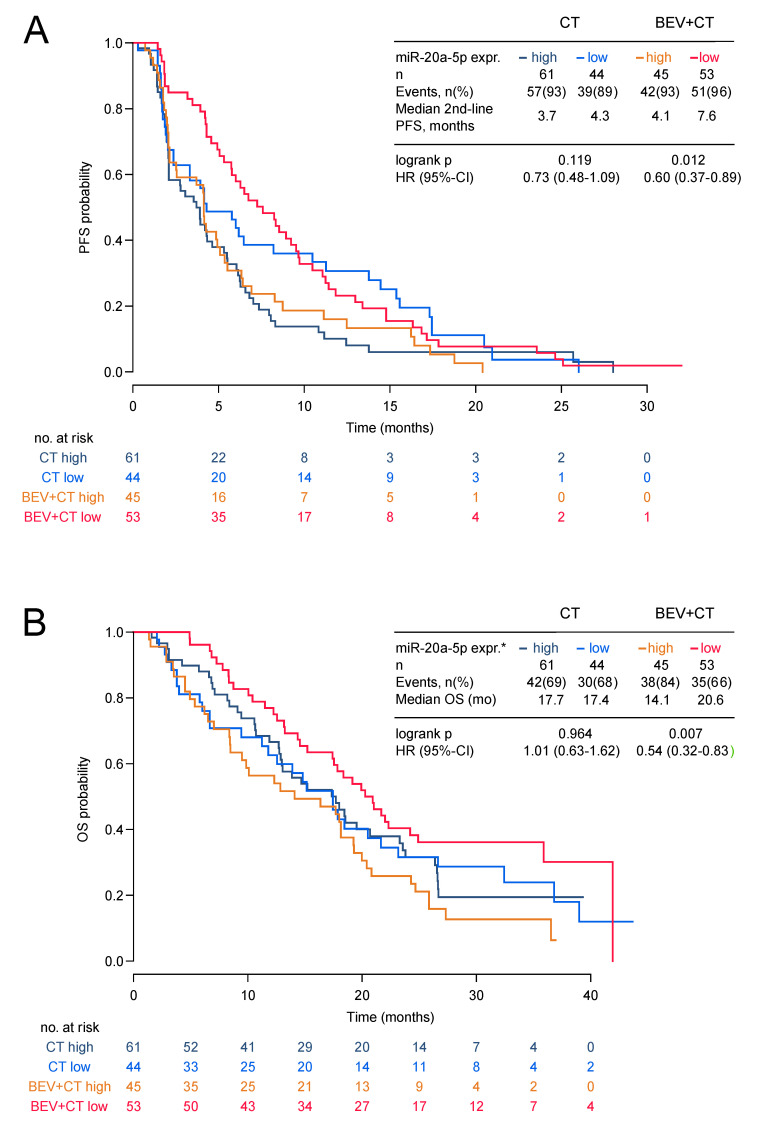
Second-line PFS and OS by miR-20a-5p expression in the TANIA phase III trial. Second-line PFS (**A**) and OS (**B**) by miR-20a-5p expression in the TANIA phase III trial (* high expression of miR-20a-5p was defined as ΔC_T_ ≤3.775 and low expression as ΔC_T_ > 3.775).

**Figure 3:**
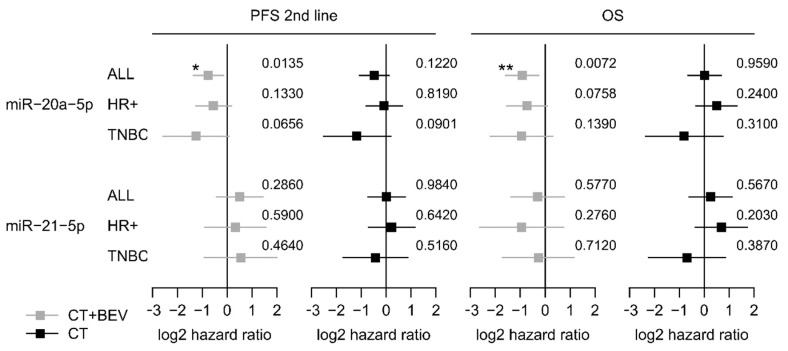
Forest plots for second-line PFS and OS based on the expression levels of miR-20a-5p and miR-21-5p in the TANIA trial. Log 2-hazard ratio (HR) and 95%-CI (forest plots) for second-line PFS and OS based on the dichtomized expression levels (low vs. high). of miR-20a-5p and miR-21-5p in the whole biomarker cohort of the TANIA trial as well as in the triple-negative and HR-positive subgroup. (*** *p* < 0.001, ** 0.001 ≤ *p* < 0.01, * 0.01 ≤ *P* < 0.05; exact *p* values on the right).

Low expression of miR-20a-5p was significantly associated with longer second-line PFS and OS in the bevacizumab arm (HR 0.60, 95%-CI 0.37–0.89; *p* = 0.012 and HR 0.54; 95%-CI 0.32–0.83; *p* = 0.007) but not in the chemotherapy alone arm (HR 0.73, 95%-CI 0.48–1.09; *p* = 0.119 and HR 1.01 95%-CI 0.63–1.62; *p* = 0.964; Figure 2a,b and Figure 3). The low miR-20a-5p expression significantly interacted also with treatment for second-line PFS and OS (*p* = 0.026; *p* = 0.007). The predictive effect of miR-20a-5p was seen both in the triple-negative subgroup and in the HR-positive subgroup in the bevacizumab arm with no significant interaction (second-line PFS *p_int_* = 0.45; OS *p_int_* = 0.61; Figure 3). For miR-21-5p no significant association with second-line PFS or OS in both treatment arms was observed (Figure S6). In multivariate analysis, the association of miR-20a-5p expression with both second-line PFS and OS remained significant (*p* = 0.037 and *p* = 0.011; Table 3).

## 4. Discussion

This is, to our knowledge, the first comprehensive analysis of miRNAs as predictive biomarkers for bevacizumab efficacy in MBC. We identified miR-20a-5p to be associated with outcome in bevacizumab treated MBC patients, which was not the case for patients without anti-VEGF treatment in first-line and second-line therapy.

The physiologic functions of most of the analyzed miRNAs are not fully known yet, especially in the context of angiogenesis. MiR-20a-5p (previous IDs: miR-20 and miR-20a) is a member of the miR-17-92 cluster, which is often dysregulated in cancer [32]. Probably depending on the cellular context, some of the cluster members induce pro-angiogenic effects [33,34]. MiR-20a-5p is downregulated by hypoxia [35], directly targets hypoxia-inducible factor (HIF)-1α, and it is contrarily upregulated by HIF-1α [36], suggesting a possible negative feedback loop. In an in vitro endothelial tube-formation assay, the mean size of the endothelial meshes was significantly increased after transfection of breast cancer cells with miR-20a-5p, whereas expression of VEGFA or other angiogenic factors was not influenced [37]. Interestingly, the effect of miR-20a-5p on vascular structure was abrogated by the VEGFA trap aflibercept, which implicates dependency on VEGFA. In renal cell carcinoma, miR-21 expression has been shown to promote cell invasiveness and angiogenesis by directly targeting the programmed cell death gene 4 (PDCD4)/c-Jun signaling pathway [38]. In an in vitro model, cytotoxic drug sensitivity of breast cancer cells was increased by miR-20a through reducing permeability glycoprotein (P-gp) mediated drug efflux controlled by a miR-20a / mitogen-activated protein kinase 1 (MAPK1) /c-Myc regulatory feedback loop [39].

Our results are counterintuitive to these findings, as a low expression but not a high expression of miR-20a was predictive for a bevacizumab effect. Therefore, we hypothesize, that miR-20a-5p contributes to a bevacizumab resistance, rather than shaping a bevacizumab responsive microenvironment by low expression of miR-20a-5p.

In two publicly available breast cancer datasets from the Total Cancer Genome Atlas (TCGA) network and The Molecular Taxonomy of Breast Cancer International Consortium (METABRIC), OS did not statistically significantly differ between miR-20a-5p low and high expression groups dichotomized by median expression levels (TCGA: HR 1.1, *p* = 0.7035; METABRIC: HR 0.84, *p* = 0.089; Figure S7) [40]. In TNBC cell lines (MDA-MB-231 and BT-20), an overexpression of miR-20a-5p has been shown to promote migration and invasion by targeting the Runt-related transcription factor 3 (RUNX3) [41]. In contrast, miR-20a-5p expression had no significant impact on survival in the TNBC subgroup of the TCGA (HR 0.42, *p =* 0.13) and METABRIC (HR 1.37, *p* = 0.21) datasets [40]. Therefore, miR-20a-5p expression seems not to be prognostic in breast cancer which is in line with our findings.

The difference in median OS between patients with low tumor expression of miR-20a-5p and patients with high tumor expression of miR-20a-5p was substantial in both bevacizumab-treated cohorts: 10.2 months in the learning set and 6.5 months in the TANIA trial, with hazard ratios of 0.46 (95%-CI 0.22–0.83) and 0.54 (95%-CI 0.32–0.83), respectively. For PFS we observed similar hazard ratios of 0.57 and 0.60 in both cohorts. Knowing that none of the randomized phase III trials in HER2 negative breast cancer comparing bevacizumab plus chemotherapy with chemotherapy alone nor meta-analyses of them showed any effect on overall survival [4,5,6,7,27,42,43,44], such a survival difference would, if confirmed, certainly influence clinical practice.

Certainly, our analysis has several limitations: first of all, the prognostic characteristics were not fully balanced between the two screening populations and several patients in the control cohort received combination chemotherapy as first-line treatment (Table 1). Both facts could explain the unexpected PFS distribution with a numerically longer PFS in the control set compared to the learning set. The differentiation between R and NR in the bevacizumab-treated learning set was based on the length of PFS, which is known to be the most sensitive parameter to assess the efficacy of a drug or combination in advanced breast cancer [45] and to correlate with OS [46]. However, patients with a PFS time close to the median overall PFS showed very small differences in miRNA expression between R and NR (Figure S2). Furthermore, the miRNA analysis in the TANIA biomarker cohort was exploratory, not preplanned and conducted retrospectively. All patients in the TANIA trial were pretreated with first-line bevacizumab. Although, a response to first-line treatment was not an inclusion criterion for this trial and in 30% of the patients first-line PFS was shorter than 6 months [26], a selection bias in favor of patients with a certain benefit to first-line bevacizumab cannot be excluded.

## 5. Conclusions

In conclusion, this is the first study providing evidence for a predictive role of tissue miRNAs for bevacizumab efficacy in metastatic breast cancer. Low miR-20a-5p expression in breast cancer tissue was predictive in two independent patient cohorts for identifying patients deriving greater benefit from bevacizumab-containing therapy.

## Figures and Tables

**Table 1:** Patient characteristics of the screening populations.

		Learning Set BEV + CT (*n* = 58)		Control Set CT Alone (*n* = 57)	
**Median age (Range)**		62	(34–81)	59	(36–86)
**DFS**	≤24 months	15	(26%)	9	(16%)
	>24 months	27	(47%)	34	(60%)
	de novo metastatic	16	(28%)	14	(25%)
**ECOG PS**	0–1	56	(97%)	51	(90%)
	2–4 and unknown	2	(3%)	7	(12%)
**Histology**	Ductal	43	(74%)	45	(79%)
	Lobular	11	(19%)	7	(12%)
	Others and unknown	4	(7%)	5	(9%)
**Grade**	1	1	(2%)	3	(5%)
	2	34	(59%)	32	(56%)
	3	22	(38%)	21	(37%)
	Unknown	1	(2%)	1	(2%)
**Receptor Status**	HR^+^/HER2^−^/G1–2	30	(52%)	24	(42%)
	HR^+^/HER2^−^/G3	11	(19%)	9	(16%)
	HR^+^/HER2^+^	2	(3%)	9	(16%)
	HR^−^/HER2^+^	1	(2%)	4	(7%)
	Triple negative	13	(22%)	10	(18%)
	HR^+^/HER2^−^/G unknown	1	(3%)	1	(2%)
**Metastases**	Visceral	37	(64%)	37	(65%)
	Non-visceral	21	(36%)	20	(35%)
**Adjuvant Endocrine Therapy**		27	(47%)	26	(46%)
**Adjuvant Chemotherapy** ^1^	Anthracycline alone	8	(14%)	10	(18%)
	Taxane alone	0	(0%)	0	(0%)
	Anthracycline and taxane	20	(35%)	6	(11%)
	No adjuvant chemotherapy	27	(47%)	35	(61%)
**Chemotherapy Backbone** ^2,3^	Paclitaxel	30	(52%)	21^4^	(37%)
	Docetaxel	5	(9%)	11^5^	(19%)
	Capecitabine	21	(36%)	9 ^6^	(16%)
	Taxane and trastuzumab	2	(3%)	10 ^7^	(18%)
	Others	0	(0%)	6 ^8^	(11%)
**Sample Type**	Primary tumor	42	(72%)	38	(67%)
	Metastasis	16	(28%)	19	(33%)
	Biopsy	22	(38%)	27	(47%)
	Resection	36	(62%)	30	(53%)
**PFS**	Number of events	54	(93%)	53	(93%)
	Median PFS (95%-CI)	10.91 (8.02–14.55)		11.79 (8.74–16.3)	
**OS**	Number of events	44	(76%)	50	(88%)
	Median OS (95%-CI)	27.3 (22.1–40.4)		23.0 (12.4–36.2)	

As the learning set and control set are not compared with each other, no *p* value is given. BEV:bevacizumab; CT: chemotherapy; DFS: disease-free survival; ECOG PS: Eastern Cooperative Oncology Group Performance Status; HR: hormone receptor; PFS: progression-free survival; OS: overall survival. ^1^ Test performed without empty categories ′taxane′ and ′de novo metastatic disease′ using Fisher’s exact test. In the control group 29 patients were treated with a chemotherapy combination. ^2,3^ Paclitaxel was combined in eight patients (14.0%) with epirubicin and in one patient (1.8%) with capecitabin. Docetaxel was combined in five patients (8.8%) with epirubicin. Capecitabine was combined in three patients (5.2%) with vinorelbine and in one patient (1.8%) with gemcitabine. Taxan and trastuzumab was combined in two patients (3.5%) with epirubicin. five Patients (8.8%) received epirubicin in combination with cyclophosphamide.

**Table 2:** Differential expression of selected miRNAs based on significantly regulated miRNAs and/or miRNAs predictive for classification between responder (R) and non-responder (NR) group using logistic regression (Least Absolute Selection and Shrinkage Operator (LASSO) regularization and 100 × 5-fold cross validation).

microRNA	Log 2FC (−ΔΔCT)	*p* Value	Adj. *p V*alue (BH) *	# LASSO/100 × 5fold CV **
hsa-miR-19b-3p	−1.46	0.00062	0.086	91
hsa-miR-21-5p	−1.56	0.00069	0.086	91
hsa-miR-9-5p	−1.95	0.00111	0.086	
hsa-miR-590-5p	−1.20	0.00124	0.086	57
hsa-miR-106b-5p	−1.08	0.00145	0.086	
hsa-miR-20a-5p	−1.53	0.00153	0.086	
hsa-miR-19a-3p	−1.60	0.00163	0.086	
hsa-miR-27a-3p	−1.28	0.00175	0.086	2
hsa-miR-210-3p	−1.12	0.00617	0.154	20
hsa-miR-224-5p	−1.42	0.01097	0.154	8
hsa-miR-155-5p	−0.93	0.01277	0.156	19
hsa-miR-28-5p	−1.07	0.01654	0.185	9

* Benjamini-Hochberg (BH) adjusted *p* value based on the false discovery rate (FDR). ** Number of times (#) microRNAs are included in the logistic regression model after LASSO regularization in 100 times of five-fold cross validation (a value of 100 indicates that this microRNA is essential for prediction). FDR: false discovery rate; LASSO: least absolute selection and shrinkage operator; CV: cross validation; log 2FC: log two-fold change.

**Table 3:** Multivariate analysis for second-line PFS and OS in the biomarker cohort of the TANIA trial.

		2nd-Line PFS			OS		
		HR	95%-CI	*p*	HR	95%-CI	*p*
miR-20a-5p	high vs. low expression	0.63	0.40–0.97	0.037	0.53	0.33–0.87	0.011
DFI	M1 vs. < 24 mo	0.82	0.34–2.00	0.670	1.17	0.43–3.24	0.760
	>24 mo vs. < 24 mo	1.18	0.67–2.08	0.570	0.53	0.27–1.05	0.068
1st-Line PFS	≥6 mo vs. < 6 mo	0.58	0.23–1.42	0.230	0.25	0.09–0.72	0.010
Histology	lobular + other vs. ductal	0.72	0.39–1.34	0.310	0.75	0.36–1.56	0.440
CT	taxane vs. non-taxane	0.89	0.45–1.76	0.730	0.70	0.29–1.67	0.430
	vinorelbine vs. non-taxane	1.38	0.76–2.50	0.290	1.10	0.57–2.14	0.780
LDH	> 1.5 ULN vs. ≤ 1.5 ULN	2.49	1.24–4.99	0.010	3.32	1.58–6.98	0.002
HR status	HR+ vs. TNBC	0.39	0.23–0.67	0.001	0.44	0.25–0.76	0.003
Metastases	visceral vs. non-visceral	1.52	0.94–2.45	0.088	0.84	0.48–1.43	0.520

DFI disease-free interval; PFS progression-free survival; OS overall survival; CT chemotherapy; LDH lactate dehydrogenase; HR hormone receptor; ULN upper limit of normal; M1: metastatic; TNBC triple negative breast cancer.

## Supplementary Materials

The following are available online at https://www.mdpi.com/2077-0383/9/6/1663/s1, Table S1: Differentially expressed miRNAs between responder (R) and non-responder (NR) group based on median PFS in the learning set; Table S2: Multivariate Cox regression analyses in the learning set for individual selected miRNAs and including risk factors as categorical covariates separately and together; Table S3: Baseline characteristics of the biomarker cohort vs ITT population (2nd-line efficacy) of the TANIA trial; Table S4: Efficacy in the biomarker cohort vs ITT population; Figure S1: TANIA trial design. Figure S2. Heatmap of normalized expression of selected miRNAs; Figure S3: Boxplots for normalized miRNA expression (ΔC_T_) in the responder group (R) vs. the non-responder group (NR). Figure S4: Kaplan-Meier curves of progression-free survival (PFS) according to expression levels of all 12 selected miRNAs in the bevacizumab treatment group and the control group. Figure S5: Kaplan-Meier curves of overall survival (OS) in the learning set according to expression levels of all 12 selected miRNAs in the bevacizumab treatment group and the control group; Figure S6: Second-line PFS and OS by miR-21-5p expression in the TANIA trial; Figure S7: Kaplan-Meier curves of overall survival (OS) in the TCGA and METABRIC datasets according to miR-20a-5p expression in breast cancer tissue.
