# Supplementary material for: Low Expression of miR-20a-5p Predicts Benefit to Bevacizumab in Metastatic Breast Cancer Patients Treated within the TANIA Phase III Trial
Source: J Clin Med. 2020 Jun 1;9(6):1663. doi: 10.3390/jcm9061663 (PMC7355487; doi:10.3390/jcm9061663)

Supplementary

Table S1. Differentially expressed miRNAs between responder (R) and non-responder (NR) group based on median PFS in the learning set.

ID	NAME	#NR	#R	NR_MEAN	R_MEAN	AVE_EXPR	LOG2FC	T	P	ADJ_P(BH)	SELECTED
396	hsa-miR-19b-3p	28	29	1,28	2,74	2,02	-1,46	-3,61	0,00062	0,086	1
397	hsa-miR-21-5p	26	28	1,71	3,27	2,52	-1,56	-3,59	0,00069	0,086	1
583	hsa-miR-9-5p	21	18	6,67	8,62	7,57	-1,95	-3,51	0,00111	0,086	1
1984	hsa-miR-590-5p	25	28	7,24	8,44	7,87	-1,20	-3,41	0,00124	0,086	1
442	hsa-miR-106b-5p	25	27	5,93	7,02	6,50	-1,08	-3,36	0,00145	0,086	1
580	hsa-miR-20a-5p	25	28	3,06	4,59	3,86	-1,53	-3,34	0,00153	0,086	1
395	hsa-miR-19a-3p	25	26	5,24	6,84	6,06	-1,60	-3,32	0,00163	0,086	1
408	hsa-miR-27a-3p	25	26	5,35	6,63	6,00	-1,28	-3,29	0,00175	0,086	0
480	hsa-miR-181a-5p	26	27	5,70	6,71	6,21	-1,01	-3,11	0,00300	0,130	0
1187	hsa-miR-140-5p	25	27	4,87	5,99	5,45	-1,12	-3,06	0,00345	0,130	0
2249	hsa-miR-143-3p	25	27	4,65	5,83	5,26	-1,18	-3,04	0,00366	0,130	0
464	hsa-miR-142-3p	25	23	5,90	7,14	6,49	-1,24	-2,97	0,00453	0,138	0
1090	hsa-miR-93-5p	25	28	3,70	4,87	4,32	-1,17	-2,96	0,00457	0,138	0
2341	hsa-miR-708-5p	25	28	6,03	7,41	6,76	-1,38	-2,92	0,00499	0,140	0
512	hsa-miR-210-3p	26	28	3,72	4,83	4,30	-1,12	-2,85	0,00617	0,154	1
2173	hsa-miR-15b-3p	22	17	8,49	9,52	8,94	-1,03	-2,85	0,00679	0,154	0
426	hsa-miR-34a-5p	25	27	4,57	5,56	5,09	-0,99	-2,75	0,00808	0,154	0
563	hsa-miR-374a-5p	25	26	6,78	7,69	7,24	-0,91	-2,74	0,00828	0,154	0
377	hsa-let-7a-5p	27	28	5,18	6,47	5,83	-1,29	-2,72	0,00858	0,154	0
2406	hsa-let-7e-5p	27	28	2,74	3,78	3,27	-1,03	-2,72	0,00866	0,154	0
1024	hsa-miR-429	28	28	5,85	7,36	6,60	-1,51	-2,71	0,00876	0,154	0
2169	hsa-miR-106a-5p	27	28	1,10	2,21	1,66	-1,11	-2,70	0,00916	0,154	0
1515	hsa-miR-660-5p	25	25	5,50	6,59	6,05	-1,09	-2,68	0,00994	0,154	0
439	hsa-miR-103a-3p	26	26	5,17	6,12	5,64	-0,95	-2,66	0,01023	0,154	0
2323	hsa-miR-454-3p	29	28	3,72	4,78	4,24	-1,06	-2,64	0,01050	0,154	0
2099	hsa-miR-224-5p	22	24	6,56	7,98	7,31	-1,42	-2,65	0,01097	0,154	1
390	hsa-miR-15b-5p	25	27	6,06	7,02	6,56	-0,96	-2,63	0,01120	0,154	0
2217	hsa-miR-18b-5p	17	18	6,71	9,57	8,18	-2,87	-2,66	0,01147	0,154	0
521	hsa-miR-218-5p	25	26	7,14	8,10	7,63	-0,96	-2,61	0,01158	0,154	0
2161	hsa-miR-324-3p	26	26	6,60	7,52	7,06	-0,91	-2,61	0,01178	0,154	0
2623	hsa-miR-155-5p	26	28	3,24	4,17	3,72	-0,93	-2,57	0,01277	0,156	1
463	hsa-miR-141-3p	27	27	4,78	5,97	5,37	-1,19	-2,57	0,01299	0,156	0
2308	hsa-miR-17-5p	27	28	1,01	2,07	1,55	-1,06	-2,56	0,01319	0,156	0

ID	NAME	#NR	#R	NR_MEAN	R_MEAN	AVE_EXPR	LOG2FC	T	P	ADJ_P(BH)	SELECTED
2084	hsa-miR-504-5p	9	15	-0,06	5,35	3,32	-5,40	-2,65	0,01352	0,156	0
411	hsa-miR-28-5p	25	24	7,16	8,24	7,69	-1,07	-2,48	0,01654	0,185	1
1516	hsa-miR-425-5p	25	28	4,29	5,18	4,76	-0,89	-2,45	0,01766	0,192	0
2428	hsa-miR-500a-5p	23	21	8,26	9,56	8,88	-1,30	-2,45	0,01822	0,193	0
507	hsa-miR-203a	25	24	5,24	6,81	6,01	-1,57	-2,42	0,01898	0,196	0
577	hsa-miR-98-5p	19	14	8,91	9,91	9,33	-1,00	-2,42	0,02062	0,206	0
2446	hsa-miR-28-3p	27	29	3,86	4,75	4,32	-0,89	-2,37	0,02135	0,206	0
2275	hsa-miR-370-3p	25	24	6,79	7,96	7,37	-1,17	-2,36	0,02236	0,206	0
433	hsa-miR-95-3p	25	26	7,68	8,55	8,13	-0,87	-2,35	0,02239	0,206	0
1518	hsa-miR-532-5p	25	25	5,83	7,04	6,43	-1,21	-2,35	0,02255	0,206	0
1149	hsa-miR-516a-3p	10	6	12,20	9,84	11,31	2,36	2,42	0,02615	0,233	0
468	hsa-miR-146a-5p	29	29	1,69	2,56	2,12	-0,88	-2,23	0,02941	0,256	0
2122	hsa-miR-376c-3p	25	24	7,16	8,02	7,58	-0,86	-2,20	0,03204	0,273	0
2412	hsa-miR-548a-5p	18	17	7,09	8,83	7,93	-1,74	-2,22	0,03281	0,274	0
2329	hsa-miR-452-5p	21	19	8,30	9,34	8,80	-1,04	-2,19	0,03375	0,276	0
1111	hsa-miR-511-5p	19	16	9,79	8,88	9,38	0,92	2,19	0,03464	0,276	0
1319	hsa-miR-374b-5p	27	28	3,83	4,89	4,37	-1,06	-2,16	0,03518	0,276	0
2304	hsa-miR-199b-3p	29	29	2,46	3,50	2,98	-1,05	-2,11	0,03858	0,289	0
2258	hsa-miR-340-5p	25	21	8,63	9,39	8,97	-0,76	-2,12	0,03884	0,289	0
457	hsa-miR-132-3p	26	27	5,21	5,92	5,57	-0,71	-2,11	0,03914	0,289	0
417	hsa-miR-30a-5p	27	28	3,75	4,80	4,28	-1,06	-2,10	0,04006	0,289	0
403	hsa-miR-25-3p	24	24	7,14	8,00	7,57	-0,86	-2,10	0,04049	0,289	0
389	hsa-miR-15a-5p	23	19	6,90	8,57	7,66	-1,67	-2,09	0,04229	0,291	0
2380	hsa-miR-106b-3p	24	22	8,03	8,81	8,40	-0,78	-2,09	0,04232	0,291	0
2438	hsa-miR-21-3p	25	22	5,55	6,42	5,96	-0,87	-2,06	0,04493	0,300	0
528	hsa-miR-301a-3p	23	19	8,64	9,56	9,06	-0,92	-2,06	0,04563	0,300	0
494	hsa-miR-195-5p	27	28	3,43	5,04	4,25	-1,62	-2,04	0,04585	0,300	0
2392	hsa-miR-301b	14	11	10,27	11,38	10,76	-1,10	-2,07	0,04789	0,304	0
2410	hsa-miR-550a-5p	14	15	9,58	10,99	10,31	-1,41	-2,06	0,04811	0,304	0
497	hsa-miR-197-3p	25	26	5,18	6,08	5,64	-0,90	-1,96	0,05477	0,341	0
407	hsa-miR-26b-5p	26	28	4,66	5,46	5,08	-0,80	-1,93	0,05891	0,359	0
2283	hsa-let-7d-5p	26	27	6,16	6,82	6,49	-0,65	-1,92	0,05954	0,359	0
539	hsa-miR-324-5p	25	20	8,10	8,78	8,40	-0,68	-1,92	0,06072	0,361	0
2285	hsa-miR-186-5p	28	29	3,89	4,59	4,25	-0,70	-1,90	0,06241	0,363	0
2278	hsa-miR-145-5p	29	29	1,49	2,29	1,89	-0,80	-1,88	0,06494	0,363	0
460	hsa-miR-135a-5p	15	10	10,39	9,31	9,96	1,07	1,92	0,06503	0,363	0

ID	NAME	#NR	#R	NR_MEAN	R_MEAN	AVE_EXPR	LOG2FC	T	P	ADJ_P(BH)	SELECTED
431	hsa-miR-92a-3p	25	27	5,50	6,18	5,85	-0,69	-1,88	0,06563	0,363	0
2100	hsa-miR-136-3p	11	8	11,30	9,67	10,62	1,63	1,94	0,06582	0,363	0
449	hsa-miR-125b-5p	26	28	3,78	4,63	4,22	-0,85	-1,87	0,06738	0,367	0
1612	hsa-miR-655-3p	16	9	10,35	9,11	9,90	1,24	1,88	0,07012	0,369	0
2282	hsa-let-7g-5p	27	28	4,32	4,89	4,61	-0,57	-1,84	0,07093	0,369	0
402	hsa-miR-24-3p	27	28	-0,46	0,16	-0,15	-0,62	-1,84	0,07095	0,369	0
435	hsa-miR-99a-5p	25	28	4,58	5,37	5,00	-0,78	-1,83	0,07270	0,369	0
2231	hsa-miR-9-3p	15	13	9,38	10,62	9,96	-1,24	-1,86	0,07306	0,369	0
2261	hsa-miR-135b-5p	23	15	7,25	8,54	7,76	-1,29	-1,84	0,07372	0,369	0
2352	hsa-miR-652-3p	26	26	6,54	7,25	6,90	-0,71	-1,82	0,07431	0,369	0
2112	hsa-miR-29a-3p	29	29	1,42	2,10	1,76	-0,68	-1,81	0,07564	0,371	0
419	hsa-miR-30c-5p	27	28	2,49	3,12	2,81	-0,63	-1,77	0,08274	0,400	0
2363	hsa-miR-202-3p	26	23	8,32	9,18	8,72	-0,85	-1,76	0,08442	0,404	0
2399	hsa-miR-582-3p	29	28	3,65	4,70	4,16	-1,05	-1,71	0,09224	0,431	0
452	hsa-miR-127-3p	26	26	6,74	7,81	7,28	-1,07	-1,71	0,09341	0,431	0
2243	hsa-miR-378	29	27	4,85	6,03	5,42	-1,19	-1,70	0,09461	0,431	0
1541	hsa-miR-548b-3p	14	11	11,29	10,18	10,80	1,11	1,73	0,09465	0,431	0
1531	hsa-miR-564	18	19	10,96	9,68	10,31	1,28	1,68	0,10086	0,454	0
2212	hsa-miR-888-5p	18	17	2,78	6,17	4,43	-3,39	-1,68	0,10189	0,454	0
554	hsa-miR-361-5p	22	22	10,19	9,18	9,68	1,01	1,66	0,10388	0,456	0
2235	hsa-miR-509-5p	26	27	3,28	4,85	4,08	-1,57	-1,65	0,10562	0,456	0
2146	hsa-miR-143-5p	17	7	10,09	8,66	9,67	1,43	1,67	0,10707	0,456	0
493	hsa-miR-194-5p	20	23	8,39	7,11	7,70	1,28	1,64	0,10784	0,456	0
2088	hsa-miR-636	26	26	8,51	9,55	9,03	-1,04	-1,63	0,10828	0,456	0
1047	hsa-miR-501-5p	15	21	7,71	8,53	8,19	-0,82	-1,64	0,10990	0,458	0
398	hsa-miR-22-3p	24	24	6,45	7,37	6,91	-0,91	-1,61	0,11340	0,468	0
2327	hsa-miR-672	11	11	7,06	10,86	8,96	-3,79	-1,63	0,11636	0,475	0
2433	hsa-miR-628-5p	29	29	6,96	7,96	7,46	-1,00	-1,59	0,11747	0,475	0
1277	hsa-miR-485-3p	18	13	9,30	10,17	9,67	-0,87	-1,59	0,12036	0,481	0
409	hsa-miR-27b-3p	26	23	7,53	8,21	7,85	-0,68	-1,56	0,12538	0,492	0
451	hsa-miR-126-5p	23	23	8,29	7,39	7,84	0,91	1,56	0,12543	0,492	0
2301	hsa-miR-22-5p	19	14	9,38	10,07	9,67	-0,68	-1,53	0,13569	0,518	0
2338	hsa-miR-483-5p	26	25	7,64	8,38	8,00	-0,74	-1,51	0,13620	0,518	0
514	hsa-miR-211-5p	12	8	11,53	10,27	11,03	1,26	1,55	0,13630	0,518	0
475	hsa-miR-152-3p	26	27	6,47	6,99	6,73	-0,52	-1,51	0,13736	0,518	0
379	hsa-let-7c-5p	27	27	6,45	7,24	6,85	-0,80	-1,50	0,13874	0,518	0

ID	NAME	#NR	#R	NR_MEAN	R_MEAN	AVE_EXPR	LOG2FC	T	P	ADJ_P(BH)	SELECTED
2409	hsa-miR-589-5p	11	7	10,60	12,78	11,45	-2,19	-1,53	0,14140	0,523	0
2174	hsa-miR-27b-5p	17	14	9,99	9,21	9,64	0,78	1,49	0,14565	0,529	0
2148	hsa-miR-144-5p	14	9	11,25	10,42	10,92	0,82	1,50	0,14631	0,529	0
2215	hsa-miR-196b-5p	29	29	5,46	6,40	5,93	-0,94	-1,46	0,14890	0,529	0
2162	hsa-miR-138-1-3p	11	9	12,17	11,11	11,69	1,06	1,49	0,15081	0,529	0
2302	hsa-miR-425-3p	23	21	9,48	8,64	9,08	0,84	1,46	0,15196	0,529	0
2293	hsa-miR-214-5p	24	25	7,82	8,51	8,17	-0,69	-1,45	0,15277	0,529	0
2172	hsa-let-7i-3p	11	9	10,33	11,15	10,70	-0,81	-1,48	0,15317	0,529	0
2403	hsa-miR-519d-3p	15	14	9,78	10,73	10,24	-0,96	-1,46	0,15459	0,529	0
1152	hsa-miR-517b	26	23	7,42	7,91	7,65	-0,49	-1,44	0,15524	0,529	0
443	hsa-miR-107	15	15	8,86	9,57	9,21	-0,71	-1,45	0,15744	0,529	0
2332	hsa-miR-409-3p	25	24	6,89	7,75	7,31	-0,85	-1,43	0,15804	0,529	0
2619	hsa-let-7b-5p	28	28	1,33	2,13	1,73	-0,80	-1,43	0,15914	0,529	0
2300	hsa-miR-200c-3p	27	28	-0,69	-0,13	-0,41	-0,56	-1,42	0,16095	0,530	0
1273	hsa-miR-362-5p	25	23	7,49	8,03	7,75	-0,54	-1,41	0,16414	0,536	0
1567	hsa-miR-604	9	14	11,33	8,95	9,88	2,38	1,42	0,16716	0,542	0
602	hsa-miR-30b-5p	27	29	3,30	3,74	3,53	-0,44	-1,39	0,17016	0,547	0
1663	hsa-miR-495-3p	20	11	9,40	9,96	9,60	-0,56	-1,38	0,17535	0,551	0
516	hsa-miR-181a-3p	19	15	9,90	10,43	10,13	-0,53	-1,38	0,17628	0,551	0
502	hsa-miR-200a-3p	26	28	5,10	5,63	5,38	-0,53	-1,37	0,17767	0,551	0
545	hsa-miR-331-3p	28	29	2,73	3,15	2,94	-0,42	-1,36	0,17787	0,551	0
2332	hsa-miR-409-3p	24	26	6,65	7,42	7,05	-0,77	-1,36	0,17855	0,551	0
2164	hsa-miR-149-3p	17	12	8,57	6,70	7,79	1,86	1,37	0,17996	0,551	0
2194	hsa-miR-886-3p	26	27	3,82	4,74	4,29	-0,92	-1,33	0,18885	0,574	0
2344	hsa-miR-556-5p	16	10	7,70	10,84	8,91	-3,14	-1,33	0,19250	0,580	0
405	hsa-miR-26a-5p	27	28	2,15	2,64	2,40	-0,49	-1,31	0,19377	0,580	0
1014	hsa-miR-20b-5p	25	25	7,49	8,04	7,77	-0,55	-1,31	0,19522	0,580	0
2279	hsa-miR-31-5p	25	29	5,31	6,06	5,71	-0,75	-1,30	0,19762	0,582	0
456	hsa-miR-130b-3p	23	21	8,29	8,72	8,50	-0,43	-1,30	0,20023	0,585	0
518	hsa-miR-215-5p	25	26	7,50	6,73	7,11	0,77	1,29	0,20144	0,585	0
2254	hsa-miR-151a-3p	28	26	4,82	5,75	5,27	-0,92	-1,29	0,20351	0,587	0
2116	hsa-miR-361-3p	12	10	7,96	10,26	9,00	-2,30	-1,29	0,20765	0,587	0
2343	hsa-miR-92b-5p	9	7	9,66	8,81	9,28	0,85	1,31	0,20775	0,587	0
387	hsa-miR-10a-5p	25	26	6,49	7,03	6,76	-0,54	-1,27	0,20836	0,587	0
2309	hsa-miR-424-3p	12	8	9,44	10,37	9,81	-0,93	-1,29	0,21089	0,587	0
2422	hsa-miR-18a-5p	18	17	9,28	9,94	9,60	-0,67	-1,27	0,21105	0,587	0

ID	NAME	#NR	#R	NR_MEAN	R_MEAN	AVE_EXPR	LOG2FC	T	P	ADJ_P(BH)	SELECTED
420	hsa-miR-30d-5p	26	27	5,02	5,62	5,32	-0,59	-1,25	0,21740	0,596	0
1138	hsa-miR-379-5p	25	22	8,98	9,59	9,26	-0,61	-1,25	0,21748	0,596	0
2005	hsa-miR-675-5p	13	10	8,68	6,45	7,71	2,23	1,25	0,22354	0,609	0
2317	hsa-miR-181a-2-3p	20	18	9,00	9,48	9,23	-0,48	-1,23	0,22624	0,612	0
2244	hsa-miR-455-3p	18	14	8,21	8,76	8,45	-0,55	-1,22	0,22993	0,617	0
587	hsa-miR-29c-3p	27	28	6,09	6,79	6,45	-0,70	-1,21	0,23209	0,618	0
2246	hsa-miR-133a-3p	26	24	7,69	8,59	8,12	-0,89	-1,21	0,23331	0,618	0
2139	hsa-miR-93-3p	25	27	5,47	5,82	5,65	-0,35	-1,20	0,23597	0,621	0
2087	hsa-miR-505-5p	19	19	9,48	9,94	9,71	-0,46	-1,19	0,24125	0,628	0
524	hsa-miR-221-3p	24	21	6,50	7,05	6,76	-0,55	-1,18	0,24412	0,628	0
508	hsa-miR-204-5p	23	19	9,10	8,57	8,86	0,53	1,17	0,24725	0,628	0
546	hsa-miR-335-5p	25	22	8,53	9,31	8,90	-0,78	-1,17	0,24857	0,628	0
1986	hsa-miR-766-3p	22	24	6,91	7,33	7,13	-0,42	-1,17	0,24863	0,628	0
2276	hsa-miR-222-3p	29	29	1,03	1,52	1,27	-0,49	-1,16	0,24905	0,628	0
436	hsa-miR-99b-5p	26	27	5,17	5,65	5,41	-0,49	-1,16	0,25011	0,628	0
529	hsa-miR-302a-3p	19	17	6,76	7,63	7,17	-0,87	-1,15	0,25609	0,639	0
1998	hsa-miR-769-5p	25	18	8,06	8,65	8,31	-0,60	-1,13	0,26457	0,653	0
2203	hsa-miR-875-5p	17	10	8,73	10,72	9,46	-1,99	-1,13	0,26857	0,653	0
2187	hsa-miR-942-5p	17	15	11,34	10,89	11,13	0,45	1,12	0,27046	0,653	0
1512	hsa-miR-657	8	8	6,61	9,71	8,16	-3,10	-1,14	0,27057	0,653	0
2274	hsa-miR-200b-5p	14	13	11,15	10,37	10,77	0,78	1,12	0,27068	0,653	0
437	hsa-miR-100-5p	25	27	4,87	5,34	5,11	-0,46	-1,11	0,27201	0,653	0
2361	hsa-miR-146b-3p	14	10	10,10	9,24	9,74	0,86	1,12	0,27315	0,653	0
527	hsa-miR-296-5p	18	14	10,89	10,21	10,59	0,68	1,11	0,27607	0,656	0
510	hsa-miR-206	8	8	7,41	9,61	8,51	-2,20	-1,11	0,27988	0,657	0
1594	hsa-miR-643	7	10	9,45	10,74	10,21	-1,29	-1,11	0,27996	0,657	0
2425	hsa-miR-19b-1-5p	15	10	10,02	10,49	10,21	-0,47	-1,09	0,28313	0,661	0
2228	hsa-miR-126-3p	29	29	-0,70	-0,36	-0,53	-0,34	-1,07	0,28740	0,666	0
2198	hsa-miR-125a-5p	26	27	5,19	5,57	5,38	-0,39	-1,07	0,28864	0,666	0
420	hsa-miR-30d-5p	26	26	4,64	5,18	4,91	-0,54	-1,06	0,29538	0,677	0
2444	hsa-miR-26b-3p	20	22	9,36	10,50	9,96	-1,14	-1,05	0,29778	0,679	0
1097	hsa-miR-146b-5p	27	29	2,01	2,35	2,19	-0,34	-1,05	0,30001	0,680	0
485	hsa-miR-184	21	19	9,34	8,72	9,05	0,62	1,04	0,30302	0,683	0
565	hsa-miR-376a-3p	25	18	9,73	10,07	9,87	-0,35	-1,03	0,30913	0,690	0
2116	hsa-miR-361-3p	14	7	9,92	10,68	10,18	-0,76	-1,04	0,30962	0,690	0
1020	hsa-miR-365b-3p	26	28	5,98	6,48	6,24	-0,50	-1,01	0,31467	0,691	0

ID	NAME	#NR	#R	NR_MEAN	R_MEAN	AVE_EXPR	LOG2FC	T	P	ADJ_P(BH)	SELECTED
592	hsa-miR-136-5p	17	9	10,43	11,34	10,75	-0,91	-1,02	0,31522	0,691	0
2183	hsa-miR-941	11	14	9,93	10,48	10,24	-0,55	-1,02	0,31563	0,691	0
1271	hsa-miR-363-3p	10	7	10,59	11,27	10,87	-0,68	-1,01	0,32536	0,709	0
1543	hsa-miR-589-3p	11	9	7,97	10,66	9,18	-2,69	-0,99	0,33065	0,715	0
2366	hsa-miR-193b-5p	16	9	9,58	9,03	9,39	0,55	0,99	0,33183	0,715	0
2434	hsa-miR-628-3p	18	13	11,45	10,82	11,19	0,63	0,96	0,34162	0,724	0
2260	hsa-miR-342-3p	28	28	1,92	1,53	1,73	0,38	0,96	0,34183	0,724	0
1280	hsa-miR-455-5p	25	18	8,21	8,56	8,36	-0,35	-0,96	0,34297	0,724	0
1553	hsa-miR-622	19	21	9,40	10,11	9,77	-0,71	-0,96	0,34412	0,724	0
2158	hsa-miR-125b-2-3p	14	9	10,30	11,06	10,60	-0,76	-0,95	0,34884	0,724	0
567	hsa-miR-378a-5p	12	11	11,06	10,55	10,82	0,51	0,95	0,34975	0,724	0
1274	hsa-miR-410-3p	21	18	10,15	10,56	10,34	-0,41	-0,94	0,35096	0,724	0
2351	hsa-miR-576-3p	12	9	9,58	11,01	10,19	-1,42	-0,95	0,35360	0,724	0
1026	hsa-miR-432-5p	15	11	8,67	9,57	9,05	-0,89	-0,94	0,35545	0,724	0
2289	hsa-miR-139-5p	26	27	7,60	7,24	7,42	0,36	0,93	0,35646	0,724	0
471	hsa-miR-148b-3p	20	16	9,34	9,81	9,55	-0,47	-0,92	0,36135	0,724	0
2251	hsa-miR-200b-3p	26	28	2,77	3,14	2,96	-0,37	-0,92	0,36190	0,724	0
2365	hsa-miR-494-3p	28	29	4,27	4,75	4,51	-0,48	-0,92	0,36239	0,724	0
1116	hsa-miR-520b	13	9	9,35	10,16	9,68	-0,81	-0,93	0,36373	0,724	0
2334	hsa-miR-182-5p	26	27	7,73	8,11	7,92	-0,37	-0,92	0,36391	0,724	0
2284	hsa-miR-138-5p	22	19	8,65	9,11	8,86	-0,46	-0,91	0,36644	0,725	0
2306	hsa-miR-214-3p	25	28	3,09	3,49	3,30	-0,39	-0,91	0,36926	0,727	0
1593	hsa-miR-618	23	19	10,19	10,60	10,38	-0,41	-0,90	0,37234	0,729	0
1279	hsa-miR-487a-3p	23	21	8,25	8,66	8,45	-0,41	-0,90	0,37466	0,729	0
533	hsa-miR-302c-3p	26	24	4,48	5,36	4,90	-0,88	-0,89	0,37684	0,729	0
2160	hsa-miR-148b-5p	12	16	10,49	10,94	10,75	-0,45	-0,90	0,37766	0,729	0
2001	hsa-miR-768-3p	9	7	11,18	10,26	10,78	0,92	0,90	0,37933	0,729	0
2222	hsa-miR-1	12	7	8,94	10,04	9,34	-1,10	-0,88	0,38647	0,736	0
2253	hsa-miR-101-3p	21	16	9,94	9,57	9,78	0,37	0,87	0,38836	0,736	0
2232	hsa-miR-135a-3p	7	11	11,07	10,27	10,58	0,80	0,88	0,38896	0,736	0
2255	hsa-miR-149-5p	26	28	5,11	5,57	5,35	-0,46	-0,86	0,39207	0,736	0
1026	hsa-miR-432-5p	12	13	9,29	9,77	9,54	-0,48	-0,87	0,39236	0,736	0
1037	hsa-miR-490-3p	14	10	10,70	10,24	10,51	0,46	0,86	0,39604	0,737	0
500	hsa-miR-199b-5p	14	9	9,59	10,10	9,79	-0,50	-0,86	0,39657	0,737	0
2423	hsa-miR-18a-3p	16	10	10,45	10,90	10,62	-0,45	-0,85	0,40135	0,741	0
2296	hsa-miR-885-5p	23	21	8,44	7,46	7,97	0,98	0,84	0,40307	0,741	0

ID	NAME	#NR	#R	NR_MEAN	R_MEAN	AVE_EXPR	LOG2FC	T	P	ADJ_P(BH)	SELECTED
2216	hsa-miR-128-3p	16	14	9,47	9,93	9,69	-0,46	-0,84	0,40478	0,741	0
2315	hsa-miR-10b-3p	23	25	7,98	8,33	8,17	-0,35	-0,84	0,40625	0,741	0
2273	hsa-miR-198	11	9	9,03	8,56	8,82	0,47	0,84	0,40975	0,744	0
2247	hsa-miR-133b	23	23	4,39	3,75	4,07	0,63	0,82	0,41453	0,747	0
2364	hsa-miR-493-3p	15	12	11,24	10,64	10,97	0,59	0,82	0,41644	0,747	0
2091	hsa-miR-513a-3p	18	15	9,60	8,60	9,14	1,00	0,82	0,42012	0,747	0
523	hsa-miR-220	24	22	8,68	8,15	8,43	0,53	0,80	0,42491	0,747	0
2214	hsa-miR-892b	7	8	11,24	10,09	10,63	1,14	0,81	0,42666	0,747	0
2447	hsa-miR-29a-5p	11	8	11,10	11,61	11,32	-0,51	-0,81	0,42697	0,747	0
2408	hsa-miR-548b-5p	11	7	11,73	11,04	11,46	0,69	0,81	0,42729	0,747	0
2184	hsa-miR-339-3p	28	28	5,97	6,32	6,15	-0,35	-0,80	0,42755	0,747	0
2263	hsa-miR-190b	21	22	8,14	8,86	8,51	-0,71	-0,80	0,42883	0,747	0
1551	hsa-miR-597-5p	11	10	12,37	11,83	12,12	0,54	0,79	0,43527	0,755	0
1818	hsa-miR-29c-5p	22	22	9,57	9,28	9,43	0,29	0,76	0,45243	0,776	0
413	hsa-miR-29b-3p	22	19	9,29	8,36	8,86	0,93	0,75	0,45517	0,776	0
2374	hsa-miR-887-3p	8	12	8,16	9,16	8,76	-1,00	-0,75	0,45954	0,776	0
2271	hsa-miR-185-5p	25	22	7,95	8,20	8,07	-0,25	-0,75	0,45966	0,776	0
2432	hsa-miR-625-3p	25	26	8,30	7,93	8,11	0,37	0,74	0,45996	0,776	0
2021	hsa-miR-674	13	15	6,40	6,96	6,70	-0,56	-0,74	0,46307	0,776	0
498	hsa-miR-199a-5p	16	12	9,71	10,12	9,89	-0,41	-0,74	0,46323	0,776	0
1186	hsa-miR-134-5p	25	26	7,48	7,76	7,62	-0,28	-0,74	0,46340	0,776	0
543	hsa-miR-328-3p	24	22	8,58	8,84	8,71	-0,26	-0,73	0,47005	0,784	0
2267	hsa-miR-545-3p	13	6	11,08	10,35	10,85	0,73	0,73	0,47567	0,790	0
2098	hsa-miR-223-5p	16	13	9,92	9,38	9,68	0,54	0,72	0,47902	0,790	0
2097	hsa-miR-222-5p	20	18	8,94	9,44	9,18	-0,50	-0,71	0,48001	0,790	0
470	hsa-miR-148a-3p	24	24	7,04	7,34	7,19	-0,30	-0,71	0,48136	0,790	0
1614	hsa-miR-572	20	17	10,64	10,07	10,38	0,57	0,70	0,48911	0,799	0
1986	hsa-miR-766-3p	21	19	6,85	7,17	7,00	-0,32	-0,69	0,49702	0,808	0
564	hsa-miR-375	25	27	6,40	5,96	6,17	0,44	0,68	0,50129	0,811	0
1184	hsa-miR-129-2-3p	7	9	9,65	10,19	9,95	-0,54	-0,68	0,50261	0,811	0
1156	hsa-miR-518b	22	24	9,11	8,45	8,76	0,66	0,67	0,50779	0,812	0
2240	hsa-miR-542-5p	23	26	6,35	5,52	5,91	0,84	0,67	0,50784	0,812	0
2237	hsa-miR-548d-5p	12	4	7,93	10,72	8,63	-2,78	-0,67	0,50964	0,812	0
2149	hsa-miR-145-3p	20	18	9,92	10,29	10,10	-0,37	-0,66	0,51459	0,813	0
1109	hsa-miR-502-5p	16	8	10,79	10,45	10,67	0,34	0,66	0,51494	0,813	0
2445	hsa-miR-27a-5p	24	21	8,85	9,10	8,97	-0,25	-0,65	0,51636	0,813	0

ID	NAME	#NR	#R	NR_MEAN	R_MEAN	AVE_EXPR	LOG2FC	T	P	ADJ_P(BH)	SELECTED
1121	hsa-miR-520g-3p	10	5	10,61	10,15	10,46	0,47	0,66	0,51853	0,813	0
553	hsa-miR-346	10	5	9,85	10,61	10,11	-0,76	-0,66	0,52058	0,813	0
2316	hsa-miR-34a-3p	23	24	9,14	8,89	9,01	0,25	0,64	0,52425	0,816	0
2002	hsa-miR-770-5p	6	11	8,98	9,81	9,52	-0,83	-0,64	0,52800	0,818	0
1621	hsa-miR-580-3p	13	8	10,23	9,74	10,04	0,50	0,63	0,53802	0,824	0
1613	hsa-miR-571	24	21	8,99	8,39	8,71	0,60	0,61	0,54306	0,824	0
2277	hsa-miR-320a	27	28	2,10	2,35	2,23	-0,25	-0,61	0,54411	0,824	0
1630	hsa-miR-491-5p	25	22	7,81	8,01	7,91	-0,20	-0,61	0,54774	0,824	0
2186	hsa-miR-345-5p	27	29	4,91	5,13	5,03	-0,22	-0,60	0,54858	0,824	0
2378	hsa-miR-125b-1-3p	12	10	10,59	10,20	10,41	0,39	0,61	0,54984	0,824	0
1550	hsa-miR-596	25	23	5,11	5,53	5,31	-0,42	-0,60	0,54989	0,824	0
2427	hsa-miR-499a-3p	13	11	8,27	7,70	8,01	0,57	0,60	0,55228	0,824	0
1039	hsa-miR-492	10	12	9,92	9,55	9,72	0,38	0,60	0,55436	0,824	0
1606	hsa-miR-661	20	18	8,37	7,97	8,18	0,40	0,60	0,55450	0,824	0
1193	hsa-miR-187-3p	16	17	7,20	7,96	7,59	-0,76	-0,60	0,55472	0,824	0
2421	hsa-miR-17-3p	11	9	10,14	10,45	10,28	-0,32	-0,59	0,56077	0,830	0
2196	hsa-miR-99b-3p	23	24	8,54	8,32	8,43	0,22	0,58	0,56438	0,832	0
489	hsa-miR-190a-5p	12	8	10,77	10,47	10,65	0,29	0,58	0,57048	0,837	0
1998	hsa-miR-769-5p	22	23	8,20	8,44	8,32	-0,24	-0,57	0,57223	0,837	0
186	hsa-miR-96-5p	13	9	10,36	10,76	10,52	-0,40	-0,57	0,57706	0,837	0
2295	hsa-miR-223-3p	26	29	2,10	2,34	2,23	-0,24	-0,56	0,57888	0,837	0
382	hsa-let-7f-5p	26	19	7,11	7,31	7,19	-0,21	-0,55	0,58181	0,837	0
2193	hsa-miR-886-5p	28	29	3,83	4,11	3,97	-0,28	-0,55	0,58479	0,837	0
2322	hsa-miR-671-3p	14	11	9,51	9,82	9,65	-0,31	-0,55	0,58615	0,837	0
2320	hsa-miR-188-5p	20	18	8,85	9,35	9,08	-0,50	-0,55	0,58692	0,837	0
2199	hsa-miR-125a-3p	21	21	10,11	9,83	9,97	0,28	0,55	0,58712	0,837	0
1558	hsa-miR-601	15	12	11,11	10,60	10,89	0,51	0,54	0,59398	0,843	0
1979	hsa-miR-431-5p	21	16	8,77	8,29	8,56	0,49	0,54	0,59554	0,843	0
1621	hsa-miR-580-3p	14	10	9,36	10,00	9,63	-0,64	-0,53	0,59786	0,843	0
2414	hsa-miR-616-3p	8	9	10,35	10,68	10,52	-0,33	-0,52	0,61003	0,853	0
2259	hsa-miR-340-3p	20	17	9,79	9,98	9,88	-0,18	-0,51	0,61124	0,853	0
1592	hsa-miR-642a-5p	16	15	10,29	10,52	10,40	-0,23	-0,51	0,61127	0,853	0
2355	hsa-miR-532-3p	25	25	7,23	6,75	6,99	0,48	0,51	0,61390	0,853	0
2388	hsa-miR-518f-3p	22	18	8,83	9,17	8,98	-0,34	-0,50	0,61897	0,857	0
1614	hsa-miR-572	16	17	10,29	9,94	10,11	0,35	0,49	0,62750	0,866	0
2236	hsa-miR-509-3p	24	22	10,28	9,98	10,14	0,30	0,48	0,63034	0,866	0

ID	NAME	#NR	#R	NR_MEAN	R_MEAN	AVE_EXPR	LOG2FC	T	P	ADJ_P(BH)	SELECTED
2083	hsa-miR-502-3p	14	12	10,09	10,33	10,20	-0,24	-0,48	0,63192	0,866	0
2156	hsa-miR-337-5p	16	10	10,28	10,02	10,18	0,26	0,48	0,63513	0,867	0
1823	hsa-miR-512-3p	17	9	10,21	9,90	10,10	0,31	0,48	0,63725	0,867	0
416	hsa-miR-30a-3p	26	26	4,72	4,98	4,85	-0,26	-0,46	0,64472	0,875	0
2141	hsa-miR-99a-3p	24	26	9,41	9,22	9,31	0,19	0,45	0,65548	0,880	0
2324	hsa-miR-744-5p	26	26	6,56	6,73	6,65	-0,17	-0,45	0,65617	0,880	0
1567	hsa-miR-604	7	10	10,88	11,32	11,14	-0,43	-0,45	0,65686	0,880	0
2227	hsa-miR-323a-3p	23	17	9,34	9,57	9,44	-0,23	-0,44	0,65919	0,880	0
454	hsa-miR-130a-3p	26	26	7,45	7,80	7,62	-0,35	-0,44	0,66024	0,880	0
2333	hsa-miR-181c-3p	12	8	9,70	9,92	9,79	-0,22	-0,44	0,66694	0,885	0
2208	hsa-miR-450b-3p	26	25	3,87	4,10	3,99	-0,23	-0,43	0,67126	0,885	0
2136	hsa-miR-33a-3p	10	12	10,18	10,39	10,29	-0,21	-0,43	0,67230	0,885	0
399	hsa-miR-23a-3p	24	20	7,42	7,90	7,64	-0,48	-0,43	0,67261	0,885	0
2270	hsa-miR-183-3p	22	22	7,93	8,09	8,01	-0,16	-0,41	0,68048	0,892	0
2386	hsa-miR-523-3p	20	13	9,88	10,34	10,06	-0,46	-0,41	0,68622	0,892	0
1550	hsa-miR-596	25	23	5,36	5,63	5,49	-0,28	-0,41	0,68639	0,892	0
1030	hsa-miR-449a	22	24	8,22	8,53	8,38	-0,31	-0,40	0,68739	0,892	0
1286	hsa-miR-539-5p	25	24	8,53	8,73	8,63	-0,20	-0,40	0,69037	0,893	0
1613	hsa-miR-571	24	21	9,09	9,43	9,25	-0,34	-0,40	0,69223	0,893	0
1544	hsa-miR-550a-3p	23	17	9,94	9,77	9,87	0,17	0,39	0,69816	0,895	0
2443	hsa-miR-26a-1-3p	16	19	10,63	10,80	10,72	-0,17	-0,39	0,70127	0,895	0
1141	hsa-miR-451a	25	22	8,08	7,88	7,98	0,20	0,39	0,70159	0,895	0
2402	hsa-miR-517b-3p	19	11	9,64	9,39	9,55	0,25	0,38	0,70561	0,895	0
1553	hsa-miR-622	20	22	9,77	9,58	9,67	0,19	0,38	0,70878	0,895	0
604	hsa-miR-424-5p	13	6	10,51	10,76	10,59	-0,25	-0,38	0,70890	0,895	0
2233	hsa-miR-331-5p	20	17	10,01	9,84	9,93	0,16	0,37	0,71278	0,895	0
1120	hsa-miR-520f-3p	14	8	10,10	10,51	10,25	-0,41	-0,37	0,71328	0,895	0
2114	hsa-miR-130b-5p	11	4	9,90	10,16	9,97	-0,26	-0,37	0,71466	0,895	0
2346	hsa-miR-551b-5p	7	8	11,35	11,02	11,18	0,33	0,37	0,71705	0,895	0
1546	hsa-miR-592	16	9	10,92	10,68	10,83	0,24	0,36	0,71919	0,895	0
2305	hsa-miR-30d-3p	18	18	10,21	10,08	10,15	0,12	0,32	0,74986	0,930	0
2269	hsa-miR-183-5p	24	21	8,66	8,79	8,72	-0,13	-0,31	0,75744	0,932	0
2434	hsa-miR-628-3p	15	17	10,88	10,72	10,79	0,17	0,31	0,76192	0,932	0
1512	hsa-miR-657	7	9	10,06	9,67	9,84	0,39	0,31	0,76259	0,932	0
2415	hsa-miR-519a-3p	19	15	9,47	9,63	9,54	-0,16	-0,30	0,76322	0,932	0
1562	hsa-miR-629-3p	23	18	10,26	10,40	10,32	-0,15	-0,30	0,76355	0,932	0

ID	NAME	#NR	#R	NR_MEAN	R_MEAN	AVE_EXPR	LOG2FC	T	P	ADJ_P(BH)	SELECTED
2436	hsa-miR-629-5p	9	9	11,00	10,78	10,89	0,22	0,30	0,76886	0,933	0
2182	hsa-miR-939-5p	25	23	5,52	5,74	5,63	-0,22	-0,30	0,76905	0,933	0
2420	hsa-miR-16-1-3p	10	12	11,26	11,03	11,13	0,24	0,29	0,77254	0,933	0
1119	hsa-miR-520e	9	9	10,39	9,94	10,16	0,45	0,29	0,77326	0,933	0
1821	hsa-miR-484	28	28	2,14	2,24	2,19	-0,10	-0,28	0,77783	0,935	0
1011	hsa-miR-200a-5p	21	21	9,47	9,59	9,53	-0,12	-0,28	0,78415	0,937	0
544	hsa-miR-330-3p	21	25	8,80	9,08	8,95	-0,28	-0,27	0,78492	0,937	0
1043	hsa-miR-497-5p	17	15	9,88	10,06	9,96	-0,18	-0,27	0,78678	0,937	0
1285	hsa-miR-487b-3p	21	14	9,88	10,01	9,93	-0,12	-0,27	0,78841	0,937	0
2431	hsa-miR-625-5p	24	23	9,46	9,35	9,41	0,11	0,26	0,79502	0,939	0
491	hsa-miR-192-5p	26	23	8,16	8,02	8,10	0,14	0,26	0,79554	0,939	0
2340	hsa-miR-423-5p	16	14	8,48	8,34	8,41	0,14	0,26	0,79955	0,941	0
400	hsa-miR-23b-3p	24	22	8,68	8,81	8,74	-0,13	-0,25	0,80245	0,941	0
2118	hsa-let-7g-3p	13	10	10,68	10,53	10,62	0,15	0,25	0,80801	0,941	0
2109	hsa-miR-32-5p	19	15	10,50	10,60	10,54	-0,10	-0,24	0,80892	0,941	0
2257	hsa-miR-339-5p	25	24	8,24	8,34	8,29	-0,10	-0,24	0,81286	0,941	0
2349	hsa-miR-574-3p	27	28	3,20	3,11	3,15	0,09	0,24	0,81398	0,941	0
2264	hsa-miR-872	11	13	11,83	11,99	11,92	-0,17	-0,24	0,81398	0,941	0
2250	hsa-miR-193a-3p	27	26	9,75	9,86	9,80	-0,11	-0,21	0,83354	0,957	0
509	hsa-miR-205-5p	25	26	5,87	5,77	5,82	0,10	0,21	0,83380	0,957	0
2299	hsa-miR-191-5p	29	29	-0,46	-0,52	-0,49	0,06	0,21	0,83676	0,957	0
1048	hsa-miR-503-5p	11	7	10,20	10,31	10,24	-0,11	-0,21	0,83740	0,957	0
2367	hsa-miR-193b-3p	28	28	2,20	2,28	2,24	-0,07	-0,20	0,84372	0,960	0
2005	hsa-miR-675-5p	11	9	8,75	8,90	8,82	-0,14	-0,19	0,84889	0,960	0
2001	hsa-miR-768-3p	11	7	10,30	10,59	10,41	-0,29	-0,19	0,85145	0,960	0
1159	hsa-miR-518d-3p	15	11	9,51	9,38	9,46	0,13	0,19	0,85203	0,960	0
1028	hsa-miR-433-3p	20	15	8,49	8,36	8,43	0,12	0,19	0,85237	0,960	0
2117	hsa-miR-362-3p	12	9	10,85	10,75	10,81	0,10	0,18	0,85710	0,960	0
1533	hsa-miR-566	15	9	10,08	10,25	10,15	-0,17	-0,18	0,85813	0,960	0
1278	hsa-miR-486-5p	26	25	5,95	5,89	5,92	0,06	0,18	0,86153	0,960	0
2419	hsa-miR-15a-3p	13	16	9,95	9,87	9,90	0,08	0,17	0,86554	0,960	0
2313	hsa-miR-139-3p	5	13	8,23	8,39	8,34	-0,15	-0,17	0,86854	0,960	0
1990	hsa-miR-758-3p	13	4	8,99	9,49	9,11	-0,50	-0,17	0,86890	0,960	0
2092	hsa-miR-508-5p	17	18	8,77	8,69	8,73	0,08	0,17	0,86952	0,960	0
1603	hsa-miR-650	23	22	6,95	7,04	7,00	-0,09	-0,16	0,87214	0,960	0
2429	hsa-miR-548am-5p	9	6	11,46	11,32	11,40	0,15	0,16	0,87510	0,960	0

ID	NAME	#NR	#R	NR_MEAN	R_MEAN	AVE_EXPR	LOG2FC	T	P	ADJ_P(BH)	SELECTED
572	hsa-miR-382-5p	24	23	9,02	8,96	8,99	0,07	0,16	0,87703	0,960	0
2185	hsa-miR-335-3p	21	20	10,07	9,99	10,03	0,08	0,15	0,87996	0,961	0
2325	hsa-miR-744-3p	11	13	10,89	10,81	10,85	0,08	0,14	0,88684	0,964	0
2398	hsa-miR-579-3p	11	6	10,81	10,72	10,78	0,09	0,14	0,88788	0,964	0
1533	hsa-miR-566	11	8	9,80	9,69	9,75	0,12	0,14	0,89245	0,964	0
1603	hsa-miR-650	24	23	7,08	7,00	7,04	0,08	0,14	0,89310	0,964	0
428	hsa-miR-34c-5p	15	5	10,42	10,52	10,45	-0,10	-0,13	0,90041	0,968	0
2147	hsa-miR-342-5p	23	19	9,50	9,57	9,53	-0,06	-0,12	0,90157	0,968	0
2297	hsa-miR-422a	26	25	8,99	8,94	8,97	0,05	0,10	0,91717	0,979	0
422	hsa-miR-30e-3p	24	26	5,83	5,78	5,80	0,05	0,10	0,92034	0,979	0
2234	hsa-miR-140-3p	27	29	6,70	6,74	6,72	-0,03	-0,09	0,92550	0,979	0
2437	hsa-miR-20a-3p	14	12	10,72	10,76	10,74	-0,04	-0,09	0,92668	0,979	0
2268	hsa-miR-874-3p	10	10	5,40	5,07	5,23	0,33	0,09	0,92757	0,979	0
482	hsa-miR-181c-5p	22	18	10,07	10,03	10,05	0,03	0,09	0,92988	0,979	0
1610	hsa-miR-411-5p	25	19	8,71	8,74	8,72	-0,03	-0,08	0,93614	0,979	0
1996	hsa-miR-454-5p	12	7	11,69	11,61	11,66	0,07	0,08	0,93757	0,979	0
473	hsa-miR-150-5p	29	29	2,26	2,22	2,24	0,04	0,08	0,93952	0,979	0
1338	hsa-miR-7-1-3p	20	20	8,89	8,93	8,91	-0,03	-0,08	0,93985	0,979	0
2395	hsa-miR-518e-3p	17	12	8,99	9,08	9,03	-0,09	-0,07	0,94183	0,979	0
1182	hsa-miR-124-3p	26	24	6,97	6,92	6,95	0,04	0,07	0,94321	0,979	0
1608	hsa-miR-449b-5p	21	21	9,24	9,20	9,22	0,05	0,07	0,94448	0,979	0
2353	hsa-miR-615-5p	8	7	10,82	10,87	10,84	-0,06	-0,06	0,95198	0,984	0
2413	hsa-miR-522-3p	28	24	5,32	5,25	5,29	0,08	0,05	0,95945	0,984	0
2218	hsa-miR-10b-5p	26	28	8,05	8,08	8,06	-0,03	-0,05	0,96106	0,984	0
2328	hsa-miR-760	14	16	8,97	9,01	8,99	-0,04	-0,05	0,96424	0,984	0
2248	hsa-miR-142-5p	20	8	10,54	10,52	10,53	0,02	0,04	0,96530	0,984	0
2093	hsa-miR-486-3p	21	22	7,77	7,79	7,78	-0,02	-0,04	0,96548	0,984	0
1043	hsa-miR-497-5p	21	17	9,71	9,73	9,72	-0,02	-0,04	0,96836	0,984	0
515	hsa-miR-212-3p	25	24	8,32	8,30	8,31	0,01	0,04	0,97074	0,984	0
2281	hsa-miR-193a-5p	25	26	7,18	7,17	7,18	0,01	0,03	0,97396	0,984	0
1153	hsa-miR-517c-3p	14	8	10,49	10,47	10,48	0,02	0,03	0,97741	0,984	0
2106	hsa-miR-188-3p	17	13	5,94	5,92	5,93	0,02	0,03	0,97803	0,984	0
1582	hsa-miR-638	18	17	10,14	10,12	10,13	0,02	0,03	0,97929	0,984	0
1988	hsa-miR-598-3p	16	11	9,93	9,92	9,93	0,01	0,02	0,98175	0,984	0
2358	hsa-miR-489-3p	26	26	7,90	7,89	7,90	0,01	0,01	0,98923	0,989	0

ID: microRNA miRBase identifier; #NR: number of non-responders expressing corresponding miRNA; #R: number of responders expressing corresponding miRNA; NR_MEAN: normalized miRNA expression (ΔCT) mean of non-responders; R_MEAN: normalized miRNA expression (ΔCT) mean of responders; AVE_EXPR: average normalized miRNA expression (ΔCT); LOG2FC: log2 fold change; T: T value; P P value; ADJ_P(BH): Benjamini-Hochberg (BH) adjusted P-value based on the false discovery rate (FDR); SELECTED: miRNA selected for validation in the validation cohort

Table S2. Multivariate Cox regression analyses in the learning set for individual selected miRNAs and including risk factors as categorical covariates separately and together (*P* < 0.05 are indicated in gray).

	**Univariate**	**Multivariate**							
	MIRNA	MIRNA+DFS	MIRNA+ADJ	MIRNA+ECOG	MIRNA+HISTO	MIRNA+GRADE	MIRNA+REC	MIRNA+METAS	MIRNA+ALL
hsa-miR-19b-3p	0,411	0,346	0,327	0,550	0,532	0,304	0,248	0,764	0,524
hsa-miR-21-5p	0,015	0,030	0,018	0,026	0,038	0,008	0,017	0,025	0,004
hsa-miR-9-5p	0,016	0,008	0,020	0,029	0,033	0,017	0,102	0,040	0,202
hsa-miR-590-5p	0,204	0,628	0,241	0,297	0,196	0,202	0,396	0,209	0,994
hsa-miR-106b-5p	0,729	0,992	0,728	0,920	0,993	0,629	0,510	0,933	0,718
hsa-miR-20a-5p	0,043	0,108	0,055	0,070	0,062	0,025	0,047	0,075	0,035
hsa-miR-19a-3p	0,259	0,514	0,307	0,371	0,344	0,113	0,226	0,271	0,114
hsa-miR-210-3p	0,045	0,029	0,035	0,073	0,107	0,028	0,075	0,132	0,219
hsa-miR-224-5p	0,005	0,022	0,002	0,009	0,029	0,005	0,152	0,007	0,087
hsa-miR-155-5p	0,138	0,078	0,140	0,209	0,154	0,178	0,060	0,288	0,067
hsa-miR-28-3p	0,134	0,159	0,134	0,201	0,242	0,118	0,235	0,306	0,507

DFS	0,069	DFS ≤ 24 months [n=15] vs.DFS > 24 months [n=27] vs. M1 at diagnosis [n=16]
ADJ	0,401	adjuvant chemotherapy (yes [n=27] vs. no [n=31])
ECOG	0,054	ECOG performance score (ECOG 0-1 [n=56] vs. ECOG ≥ 2 [n=2])
HISTO	0,144	histologic subtype (ductal [n=43] vs. lobular[n=11] vs. others [n=3])
GRADE	0,258	grade (1-2 [n=35] vs. 3 [n=22])
REC	0,134	receptor status (hormone receptor positive/HER2 negative [n=42] vs. HER2 positive [n=3] vs. triple-negative [n=13])
METAS	0,049	location of metastases (visceral [n=21] vs. non-visceral [n=37])

Table S3. Baseline characteristics of the biomarker cohort vs ITT population (2nd-line efficacy) of the TANIA trial.

**Characteristic**	**TANIA biomarker cohort**		**TANIA ITT population**	
	**CT alone (*N* = 105)**	**BEV + CT (*N* = 98)**	**CT alone (*N* = 247)**	**BEV + CT (*N* = 247)**
**TNBC**	33 (31%)	25 (26%)	57 (23%)	49 (20%)
**HR-positive**	72 (69%)	73 (74%)	188 (76%)	198 (80%)
**1st-line PFS < 6 m**	16 (15%)	8 (8%)	32 (13%)	25 (10%)

**Choice of CT**				
Taxane	16 (15%)	12 (12%)	32(13%)	32 (13%)
Non-taxane	79 (75%)	70 (72%)	191 (77%)	188 (76%)
Vinorelbine	10 (10%)	16 (16%)	24 (10%)	27 (11%)
**LDH > 1.5 ULN**	23 (22%)	14 (14%)	40 (16%)	37 (15%)
**Age, years** Median (range)	53 (30-76)	55 (24-80)	54 (30-77)	56 (24-81)
**DFI ≤ 24 months**	24 (23%)	21 (21%)	58 (23%)	53 (21%)
**≥3 metastatic sites**	36 (34%)	23 (23%)	88 (36%)	80 (32%)
**Visceral metastases**	76 (72%)	70 (71%)	190 (77%)	186 (75%)
**Liver metastases**	65 (62%)	54 (55%)	151 (61%)	143 (58%)

TNBC: triple-negative breast cancer; HR: hormone receptor; PFS: progression-free survival; LDH: lactate dehydrogenase; ULN: upper limit of normal; DFI: disease-free interval; BEV: bevacizumab; CT: chemotherapy; ITT: intent-to-treat;

Table S4. Efficacy in the biomarker cohort vs ITT population.

**Endpoint**	**TANIA biomarker cohort**		**TANIA ITT population**	
	**CT alone (*N* = 105)**	**BEV + CT (*N* = 98)**	**CT alone (*N* = 247)**	**BEV + CT (*N* = 247)**
**2nd-line PFS**				
Events, n (%)	96 (91%)	93 (98%)	203 (82%)	204 (83%)
Median, months (95%CI)	3.9 (2.8-5.8)	5.4 (4.3-7.3)	4.2 (3.9-4.7)	6.3 (5.4-7.2)
Stratified HR (95%CI)	**0.86** (0.65-1.15)		**0.75** (0.61-0.93)	
log-rank p-value	0.310		0.007	
**OS**				
Events, n (%)	72 (69%)	73 (74%)	156 (63%)	163 (66%)
Median, months (95%CI)	17.4 (13.0-20.7)	18.1 (14.6-20.9)	18.7 (15.4-21.2)	19.7 (17.6-21.0)
Stratified HR (95%CI)	**0.95** (0.69-1.32)		**0.96** (0.76-1.21)	
log-rank p-value	0.745		0.725	

PFS: progression-free survival; CI: confidence interval; HR: hazard ratio; OS: overall survival; BEV: bevacizumab; CT: chemotherapy; ITT: intent-to-treat;

Figure S1. TANIA trial design

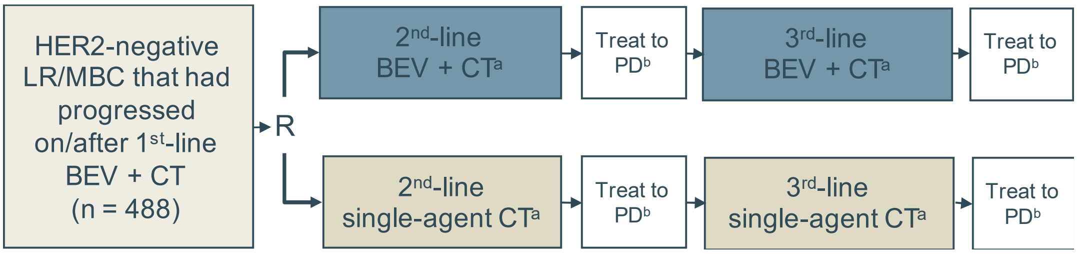

Stratification factors:

- Hormone receptor status
- Time to first progression (<6 vs. ≥ 6 months)
- Choice of CT (taxane vs. non-taxane vs. vinorelbine)
- LDH concentration (≤ 1.5 vs. > 1.5 x ULN)

[a] Choice of CT at investigator's discretion. [b] Or unacceptable toxicity or withdrawal of consent. LDH lactate dehydrogenase; PD progressive disease; ULN upper limit of normal.

Figure S2. Heatmap of mean centered expression of selected miRNAs (blue down regulation and red upregulation compared to the respective mean; log2 fold changes are color coded according to the legend). Progression free survival (PFS) of the respective patients is shown as bars, gray means progression occurred and light-grey progression was not observed (censored data).

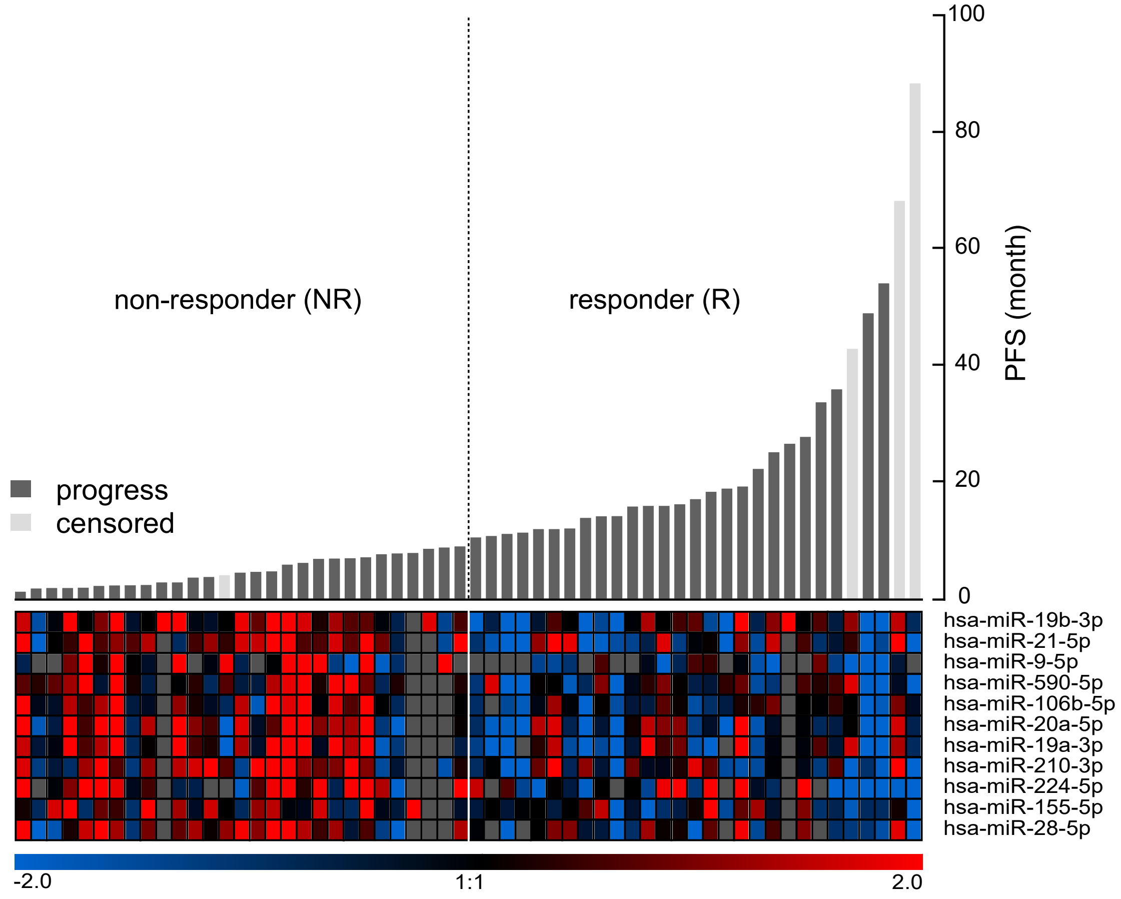

Figure S3. Boxplots for normalized miRNA expression (ΔC_T) in the responder group (R) vs. the non-responder group (NR). Note: high ΔC_T levels means lower expression and low ΔC_T levels means higher expression. P-values were adjusted based on the false discovery rate (FDR) according to Benjamini-Hochberg method and provided in brackets.

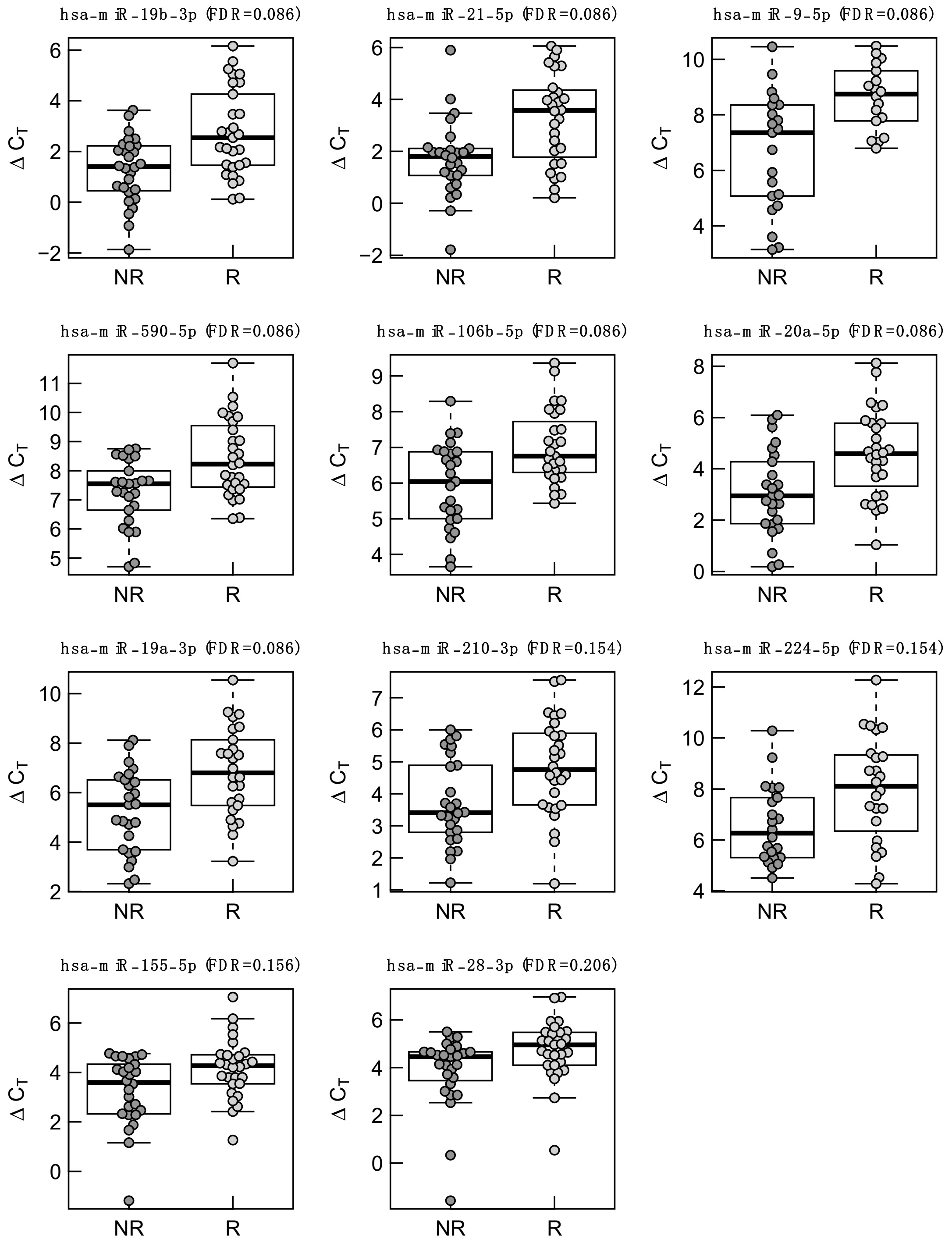

Figure S4. Kaplan-Meier curves of progression-free survival (PFS) according to expression levels of all 12 selected miRNAs in the bevacizumab treatment group and the control group. Patients are dichotomized based on median expression in the respective group. *P*-values were calculated with log-rank test.

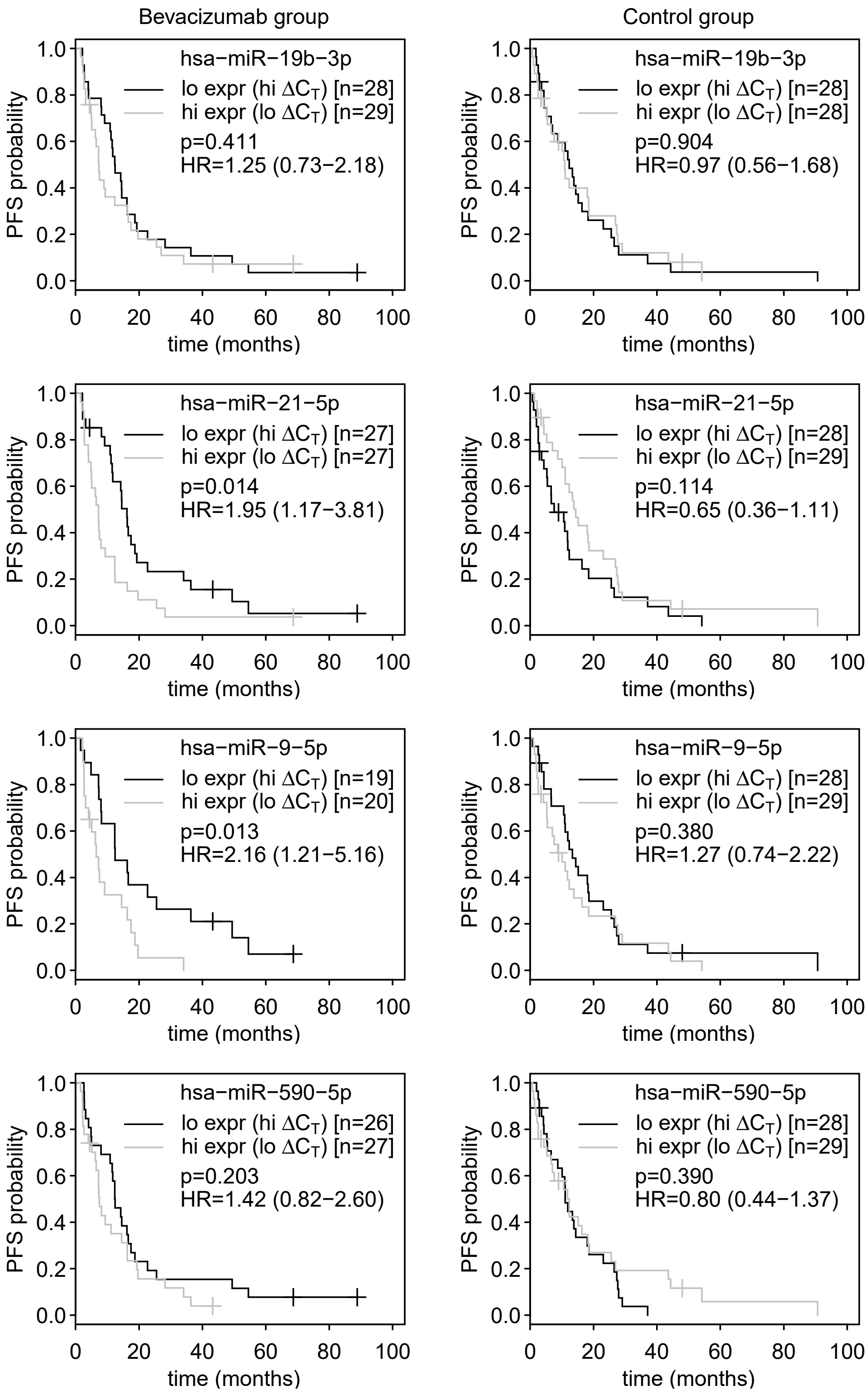
Bevacizumab group
Control group
hsa−miR−19b−3p
lo expr (hi ΔC_T) [n=28]
hi expr (lo ΔC_T) [n=29]
p=0.411
HR=1.25 (0.73−2.18)
hsa−miR−19b−3p
lo expr (hi ΔC_T) [n=28]
hi expr (lo ΔC_T) [n=28]
p=0.904
HR=0.97 (0.56−1.68)
hsa−miR−21−5p
lo expr (hi ΔC_T) [n=27]
hi expr (lo ΔC_T) [n=27]
p=0.014
HR=1.95 (1.17−3.81)
hsa−miR−21−5p
lo expr (hi ΔC_T) [n=28]
hi expr (lo ΔC_T) [n=29]
p=0.114
HR=0.65 (0.36−1.11)
hsa−miR−9−5p
lo expr (hi ΔC_T) [n=19]
hi expr (lo ΔC_T) [n=20]
p=0.013
HR=2.16 (1.21−5.16)
hsa−miR−9−5p
lo expr (hi ΔC_T) [n=28]
hi expr (lo ΔC_T) [n=29]
p=0.380
HR=1.27 (0.74−2.22)
hsa−miR−590−5p
lo expr (hi ΔC_T) [n=26]
hi expr (lo ΔC_T) [n=27]
p=0.203
HR=1.42 (0.82−2.60)
hsa−miR−590−5p
lo expr (hi ΔC_T) [n=28]
hi expr (lo ΔC_T) [n=29]
p=0.390
HR=0.80 (0.44−1.37)
PFS probability
time (months)
0.0
0.2
0.4
0.6
0.8
1.0
0
20
40
60
80
100

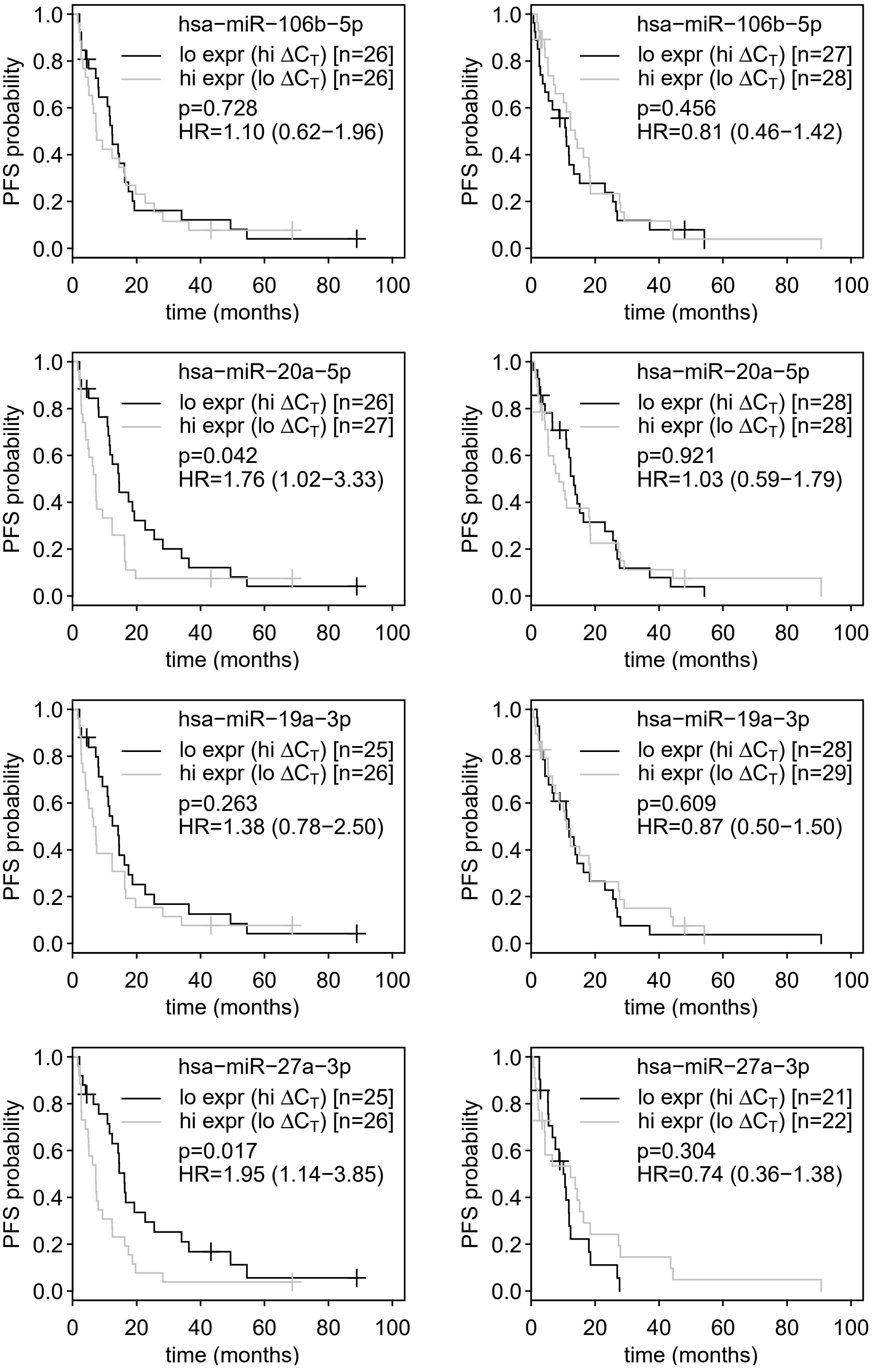
hsa−miR−106b−5p
lo expr (hi ΔC_T) [n=26]
hi expr (lo ΔC_T) [n=26]
p=0.728
HR=1.10 (0.62−1.96)
hsa−miR−106b−5p
lo expr (hi ΔC_T) [n=27]
hi expr (lo ΔC_T) [n=28]
p=0.456
HR=0.81 (0.46−1.42)
hsa−miR−20a−5p
lo expr (hi ΔC_T) [n=26]
hi expr (lo ΔC_T) [n=27]
p=0.042
HR=1.76 (1.02−3.33)
hsa−miR−20a−5p
lo expr (hi ΔC_T) [n=28]
hi expr (lo ΔC_T) [n=28]
p=0.921
HR=1.03 (0.59−1.79)
hsa−miR−19a−3p
lo expr (hi ΔC_T) [n=25]
hi expr (lo ΔC_T) [n=26]
p=0.263
HR=1.38 (0.78−2.50)
hsa−miR−19a−3p
lo expr (hi ΔC_T) [n=28]
hi expr (lo ΔC_T) [n=29]
p=0.609
HR=0.87 (0.50−1.50)
hsa−miR−27a−3p
lo expr (hi ΔC_T) [n=25]
hi expr (lo ΔC_T) [n=26]
p=0.017
HR=1.95 (1.14−3.85)
hsa−miR−27a−3p
lo expr (hi ΔC_T) [n=21]
hi expr (lo ΔC_T) [n=22]
p=0.304
HR=0.74 (0.36−1.38)
PFS probability
time (months)
0.0 0.2 0.4 0.6 0.8 1.0
0 20 40 60 80 100

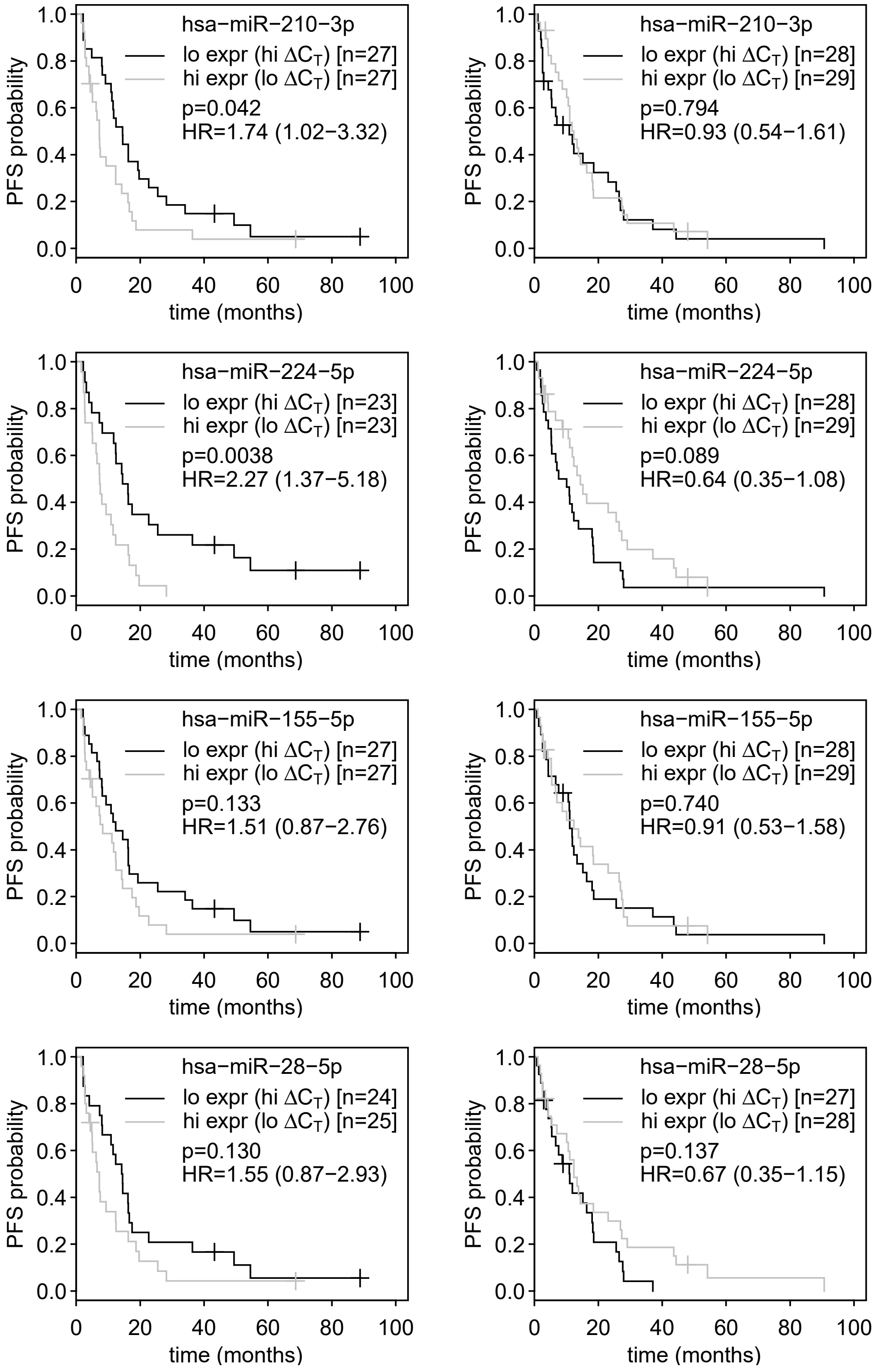

Figure S5. Kaplan-Meier curves of overall survival (OS) in the learning set according to expression levels of all 12 selected miRNAs in the bevacizumab treatment group and the

control group. Patients are dichotomized based on median expression in the respective group. *P*-values were calculated with log-rank test.

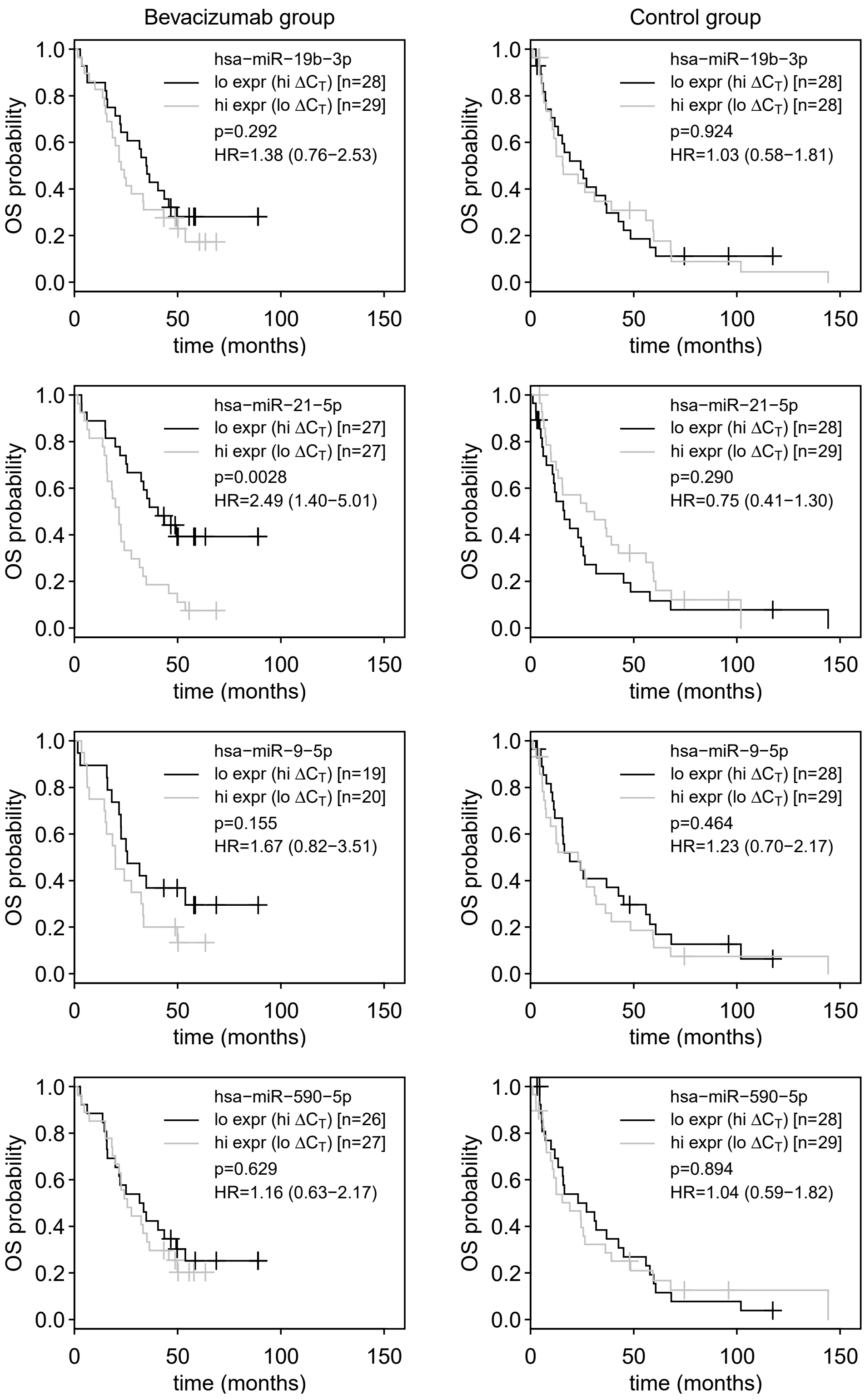

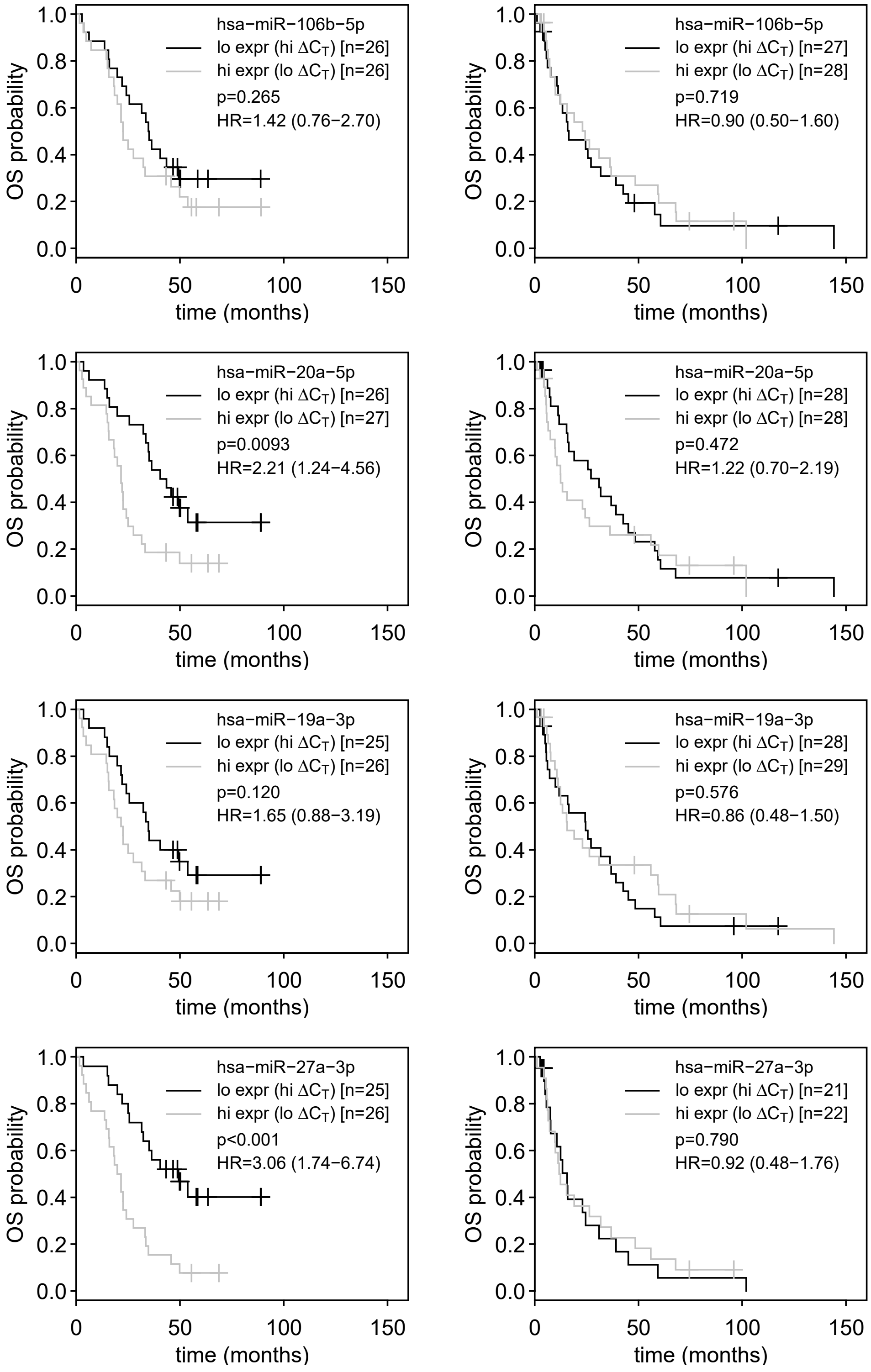
hsa−miR−106b−5p
lo expr (hi ΔC$_T$) [n=26]
hi expr (lo ΔC$_T$) [n=26]
p=0.265
HR=1.42 (0.76−2.70)
hsa−miR−106b−5p
lo expr (hi ΔC$_T$) [n=27]
hi expr (lo ΔC$_T$) [n=28]
p=0.719
HR=0.90 (0.50−1.60)
hsa−miR−20a−5p
lo expr (hi ΔC$_T$) [n=26]
hi expr (lo ΔC$_T$) [n=27]
p=0.0093
HR=2.21 (1.24−4.56)
hsa−miR−20a−5p
lo expr (hi ΔC$_T$) [n=28]
hi expr (lo ΔC$_T$) [n=28]
p=0.472
HR=1.22 (0.70−2.19)
hsa−miR−19a−3p
lo expr (hi ΔC$_T$) [n=25]
hi expr (lo ΔC$_T$) [n=26]
p=0.120
HR=1.65 (0.88−3.19)
hsa−miR−19a−3p
lo expr (hi ΔC$_T$) [n=28]
hi expr (lo ΔC$_T$) [n=29]
p=0.576
HR=0.86 (0.48−1.50)
hsa−miR−27a−3p
lo expr (hi ΔC$_T$) [n=25]
hi expr (lo ΔC$_T$) [n=26]
p<0.001
HR=3.06 (1.74−6.74)
hsa−miR−27a−3p
lo expr (hi ΔC$_T$) [n=21]
hi expr (lo ΔC$_T$) [n=22]
p=0.790
HR=0.92 (0.48−1.76)
OS probability
time (months)
0.0
0.2
0.4
0.6
0.8
1.0
0
50
100
150

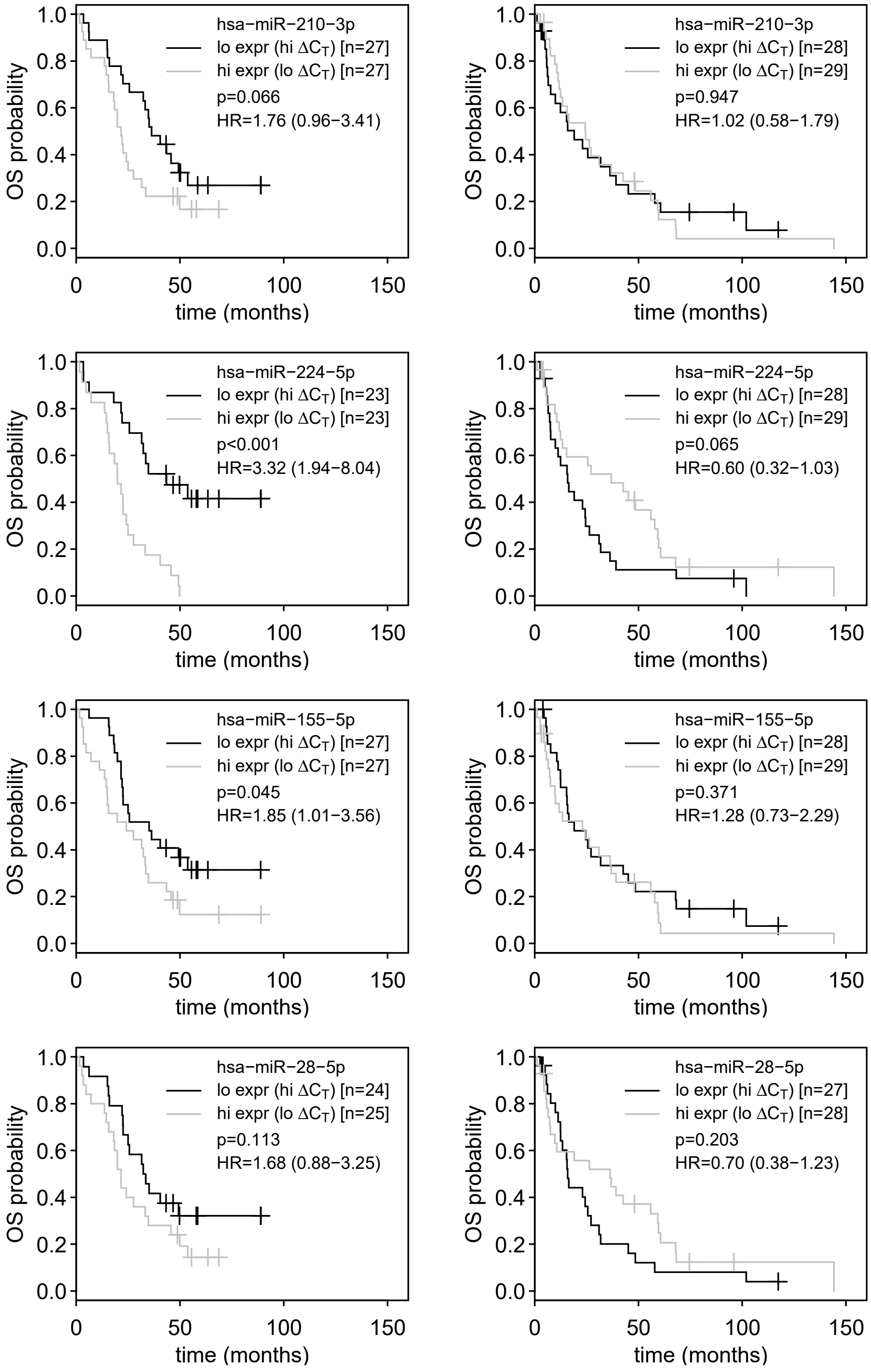
hsa−miR−210−3p
lo expr (hi ΔC_T) [n=27]
hi expr (lo ΔC_T) [n=27]
p=0.066
HR=1.76 (0.96−3.41)
hsa−miR−210−3p
lo expr (hi ΔC_T) [n=28]
hi expr (lo ΔC_T) [n=29]
p=0.947
HR=1.02 (0.58−1.79)
hsa−miR−224−5p
lo expr (hi ΔC_T) [n=23]
hi expr (lo ΔC_T) [n=23]
p<0.001
HR=3.32 (1.94−8.04)
hsa−miR−224−5p
lo expr (hi ΔC_T) [n=28]
hi expr (lo ΔC_T) [n=29]
p=0.065
HR=0.60 (0.32−1.03)
hsa−miR−155−5p
lo expr (hi ΔC_T) [n=27]
hi expr (lo ΔC_T) [n=27]
p=0.045
HR=1.85 (1.01−3.56)
hsa−miR−155−5p
lo expr (hi ΔC_T) [n=28]
hi expr (lo ΔC_T) [n=29]
p=0.371
HR=1.28 (0.73−2.29)
hsa−miR−28−5p
lo expr (hi ΔC_T) [n=24]
hi expr (lo ΔC_T) [n=25]
p=0.113
HR=1.68 (0.88−3.25)
hsa−miR−28−5p
lo expr (hi ΔC_T) [n=27]
hi expr (lo ΔC_T) [n=28]
p=0.203
HR=0.70 (0.38−1.23)
OS probability
time (months)
0.0
0.2
0.4
0.6
0.8
1.0
0
50
100
150

Figure S6. Second-line PFS (A) and OS (B) by miR-21-5p expression in the TANIA trial. (*high expression of miR-21a-5p was defined as ΔCT≤ 2.080 and low expression as ΔCT>2.080).

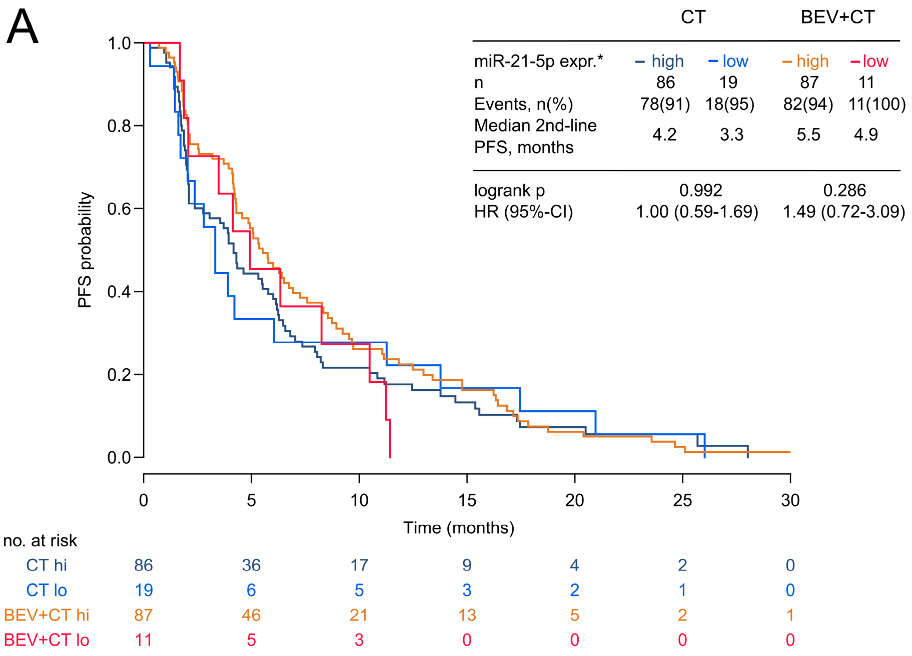

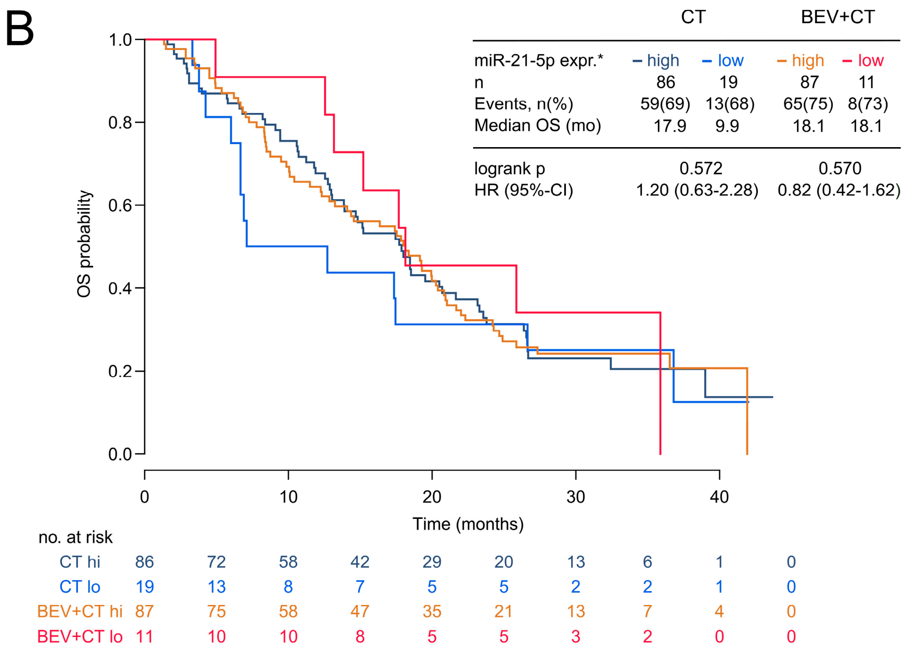

Figure S7. Kaplan-Meier curves of overall survival (OS) in the (A) TCGA and (B) METABRIC datasets according to miR-20a-5p expression in breast cancer tissue dichotomized by median expression levels. In both datasets, all data were included without restriction according to clinical or molecular characteristics.

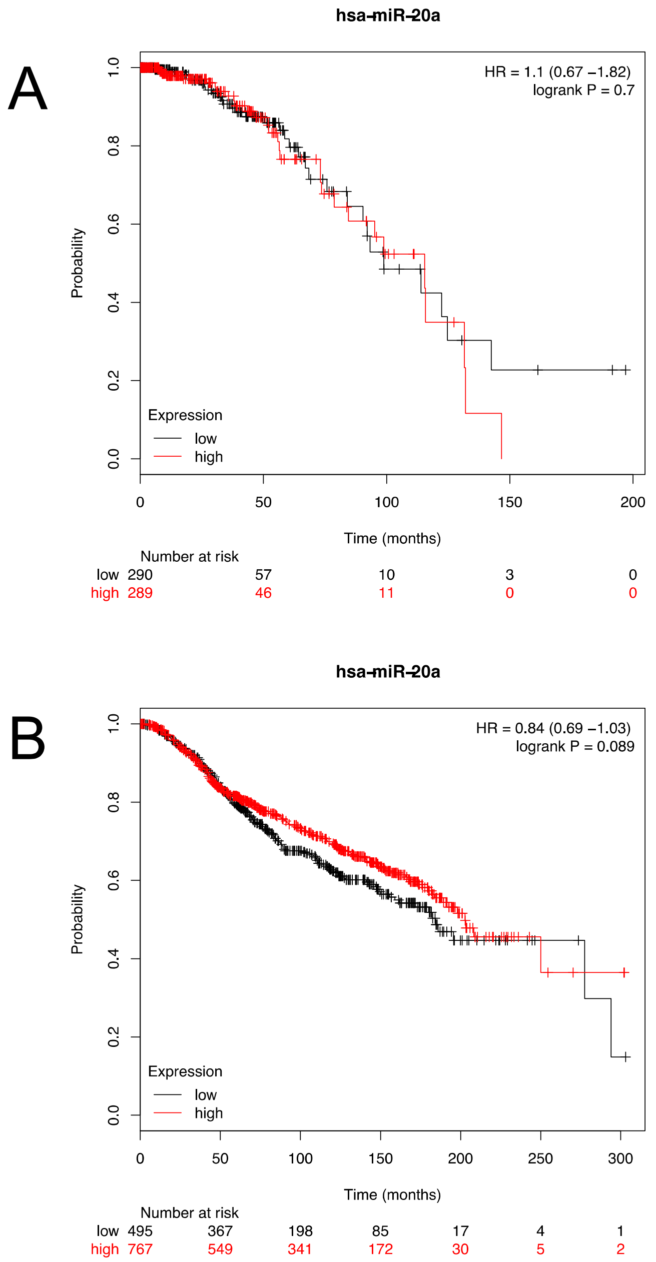

Supplement: Supplementary file 1 [file jcm-09-01663-s001.pdf]
